# A Comprehensive Map of Insulator Elements for the *Drosophila* Genome

**DOI:** 10.1371/journal.pgen.1000814

**Published:** 2010-01-15

**Authors:** Nicolas Nègre, Christopher D. Brown, Parantu K. Shah, Pouya Kheradpour, Carolyn A. Morrison, Jorja G. Henikoff, Xin Feng, Kami Ahmad, Steven Russell, Robert A. H. White, Lincoln Stein, Steven Henikoff, Manolis Kellis, Kevin P. White

**Affiliations:** 1 Institute for Genomics and Systems Biology, Department of Human Genetics, and Department of Ecology and Evolution, University of Chicago, Chicago, Illinois, United States of America; 2 Computer Science and Artificial Intelligence Lab, Massachusetts Institute of Technology, Cambridge, Massachusetts, United States of America; 3 Division of Basic Sciences, Fred Hutchinson Cancer Research Center, Seattle, Washington, United States of America; 4 Howard Hughes Medical Institute, Seattle, Washington, United States of America; 5 Department of Biomedical Engineering, State University of New York at Stony Brook, Stony Brook, New York, United States of America; 6 Department of Biological Chemistry and Molecular Pharmacology, Harvard Medical School, Boston, Massachusetts, United States of America; 7 Department of Genetics, University of Cambridge, Cambridge, United Kingdom; 8 Department of Physiology, Development, and Neuroscience, University of Cambridge, Cambridge, United Kingdom; 9 Ontario Institute for Cancer Research, Toronto, Canada; 10 Cold Spring Harbor Laboratory, Cold Spring Harbor, New York, United States of America; 11 Broad Institute, Massachusetts Institute of Technology and Harvard University, Cambridge, Massachusetts, United States of America; RIKEN Genomic Sciences Center, Japan

## Abstract

Insulators are DNA sequences that control the interactions among genomic regulatory elements and act as chromatin boundaries. A thorough understanding of their location and function is necessary to address the complexities of metazoan gene regulation. We studied by ChIP–chip the genome-wide binding sites of 6 insulator-associated proteins—dCTCF, CP190, BEAF-32, Su(Hw), Mod(mdg4), and GAF—to obtain the first comprehensive map of insulator elements in *Drosophila* embryos. We identify over 14,000 putative insulators, including all classically defined insulators. We find two major classes of insulators defined by dCTCF/CP190/BEAF-32 and Su(Hw), respectively. Distributional analyses of insulators revealed that particular sub-classes of insulator elements are excluded between *cis*-regulatory elements and their target promoters; divide differentially expressed, alternative, and divergent promoters; act as chromatin boundaries; are associated with chromosomal breakpoints among species; and are embedded within active chromatin domains. Together, these results provide a map demarcating the boundaries of gene regulatory units and a framework for understanding insulator function during the development and evolution of *Drosophila*.

## Author Summary

The spatiotemporal specificity of gene expression is controlled by interactions among regulatory proteins, *cis*-regulatory elements, chromatin modifications, and genes. These interactions can occur over large distances, and the mechanisms by which they are controlled are poorly understood. Insulators are DNA sequences that can both block the interaction between regulatory elements and genes, as well as block the spread of regions of modified chromatin. To date, relatively few insulators have been identified in developing *Drosophila* embryos. We here present the genome wide identification of over 14,000 binding sites for 6 insulator-associated proteins. We demonstrate the existence of two broad classes of insulators. Insulators of both classes are enriched at the boundaries of a particular chromatin modification. However, only insulators bound by BEAF-32, CP190, and dCTCF are enriched in regions of open chromatin or demarcate gene boundaries, with a particular enrichment between differentially expressed promoters. Furthermore, insulators of this class are enriched at points of chromosomal rearrangement among the 12 species of sequenced *Drosophila*, suggesting that insulator defined regulatory boundaries are evolutionarily conserved.

## Introduction

The spatiotemporal regulation of transcription is controlled by the binding of transcription factors to their target *cis*-regulatory modules (CRM) and their resulting interactions with promoters. Such regulatory interactions between CRMs and promoters can occur over short distances when regulatory sequences are immediately proximal to their target promoter or, in many cases, over longer distances involving many thousands of base pairs. Because of the variability in the distances over which CRMs can act, delineating the molecular boundaries of genes can be challenging. Mechanisms by which a CRM targets the appropriate promoter among a collection of adjacent promoters are poorly defined. However, one such mechanism involves the partitioning of the genome into regulatory domains by genome features known as insulators, or boundary DNA elements.

Since their initial characterization twenty years ago [1]–[4], insulator elements have been thought to create distinct regulatory domains, and thus allow enhancers to find their proper target promoter [5]. Insulators have been identified in *Drosophila* as well as in vertebrate genomes [6] based on their ability to disrupt the communication between an enhancer and a promoter when inserted between them. This enhancer-blocking activity is dependent upon the binding of insulators by several proteins. The CCCTC-binding Factor (CTCF) was first identified in vertebrates [7]; its *Drosophila* homolog, dCTCF, is known to bind several insulators and is necessary for their function [8]–[11]. CTCF is currently the only vertebrate protein known to be associated with insulator elements. In *Drosophila* however, several other proteins have been identified for their insulator function. Su(Hw) is associated with the *gypsy* retrotransposon insulator and other endogenous binding sites [4], [12]–[14]. The insulator activity of *gypsy* is dependent on the recruitment by Su(Hw) of two other proteins: Modifier of mdg4 [Mod(mdg4)] [15],[16] and CP190 [17]. Three additional proteins have been linked to insulator function in *Drosophila*. The binding of Zw5 and BEAF-32 on the scs/scs' elements of the hsp70 locus is required for their enhancer-blocking activity [18],[19]. Similarly, the ubiquitous transcription factor GAF (GAGA Associated Factor) is necessary for the enhancer-blocking activity of particular insulators [20]–[22]. Apart from their enhancer-blocking activity, insulators act as chromatin boundary elements. Such boundaries block the spreading of epigenetic marks or chromatin proteins such as repressive heterochromatin proteins or Polycomb Group-dependent (PcG) silencing [23]–[27].

While genetic and molecular studies of insulator function suggest that insulators play a major role in the regulatory organization of the genome, functional data have been collected on only a dozen insulator sequences in *Drosophila* and mammals. The identification of new insulators in flies and mammals by genome-wide approaches has only recently been initiated in different biological sources [10], [14], [27]–[30]. Here we provide a uniformly collected dataset and comprehensive analysis from developing embryos for six different insulator proteins.

## Results

### Genome-wide mapping of insulator-associated proteins

**Figure 1:**
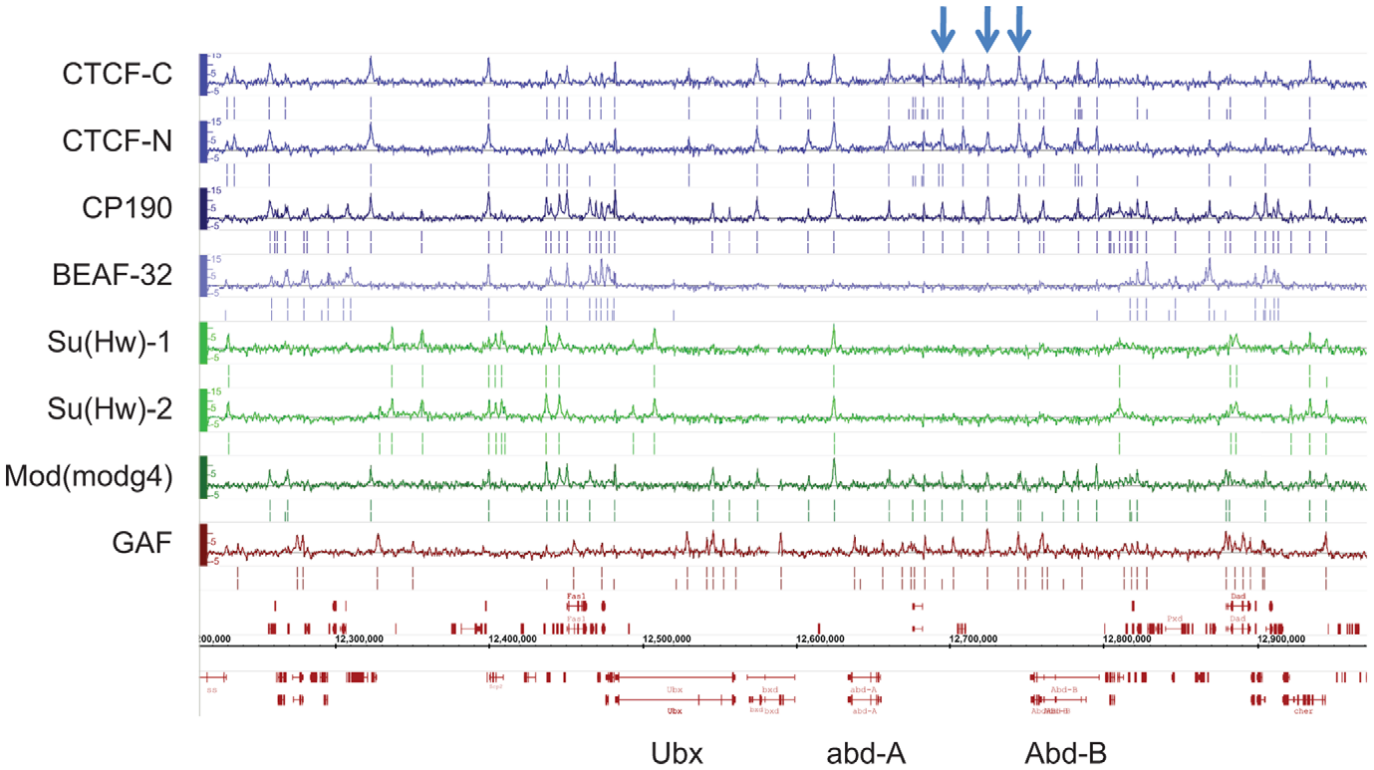
Binding profile of insulator-associated proteins in *Drosophila*. Binding profile of the 6 proteins studied on a large region of the chromosome 3R including the Bithorax-Complex, which contains 3 well-characterized insulators (blue arrows). For each protein, the track depicts the MAT score of each probe plotted on the y-axis versus chromosomal position, plotted along the x-axis. Called binding sites at 1% and 5% FDR are marked below each track. Note CTCF-C/CTCF-N and Su(Hw)-1/Su(Hw)-2 represent data from 2 independent antibodies. Flybase annotated genes are represented in red as the two bottom tracks.

We mapped the genome-wide binding sites of 6 insulator-associated proteins: CTCF, CP190, BEAF-32, Su(Hw), Mod(mdg4) and GAF by Chromatin ImmunoPrecipitation coupled with microarrays (ChIP-chip) in *Drosophila* embryos (0–12 h of development). For CTCF and Su(Hw), 2 different antibodies for each factor were used as controls to demonstrate the reproducibility of our experiments. At a 1% False Discovery Rate (FDR), we identified between 2,500 and 6,600 binding sites for each factor (Figure 1 and Table S1), which included all functionally verified *Drosophila melanogaster* insulator sequences (Figure 1, Table S2, and Figure S1). The reproducibility of different ChIP-chip experiments for 2 different antibodies for CTCF and Su(Hw) is very high, with 94% of CTCF and 87% of Su(Hw) binding sites overlapping (Figure S2 and Figure S3). Moreover, we were able to recapitulate the profiles for CTCF and Su(Hw) generated in *Drosophila* embryos for the homeotic complexes and 3 Mb of the *Adh* region [10],[14] with an overlap of 94% (31/33) for CTCF and 70% (27/41) for Su(Hw) between the published dataset and our mapping in the same genomic region.

**Figure 2:**
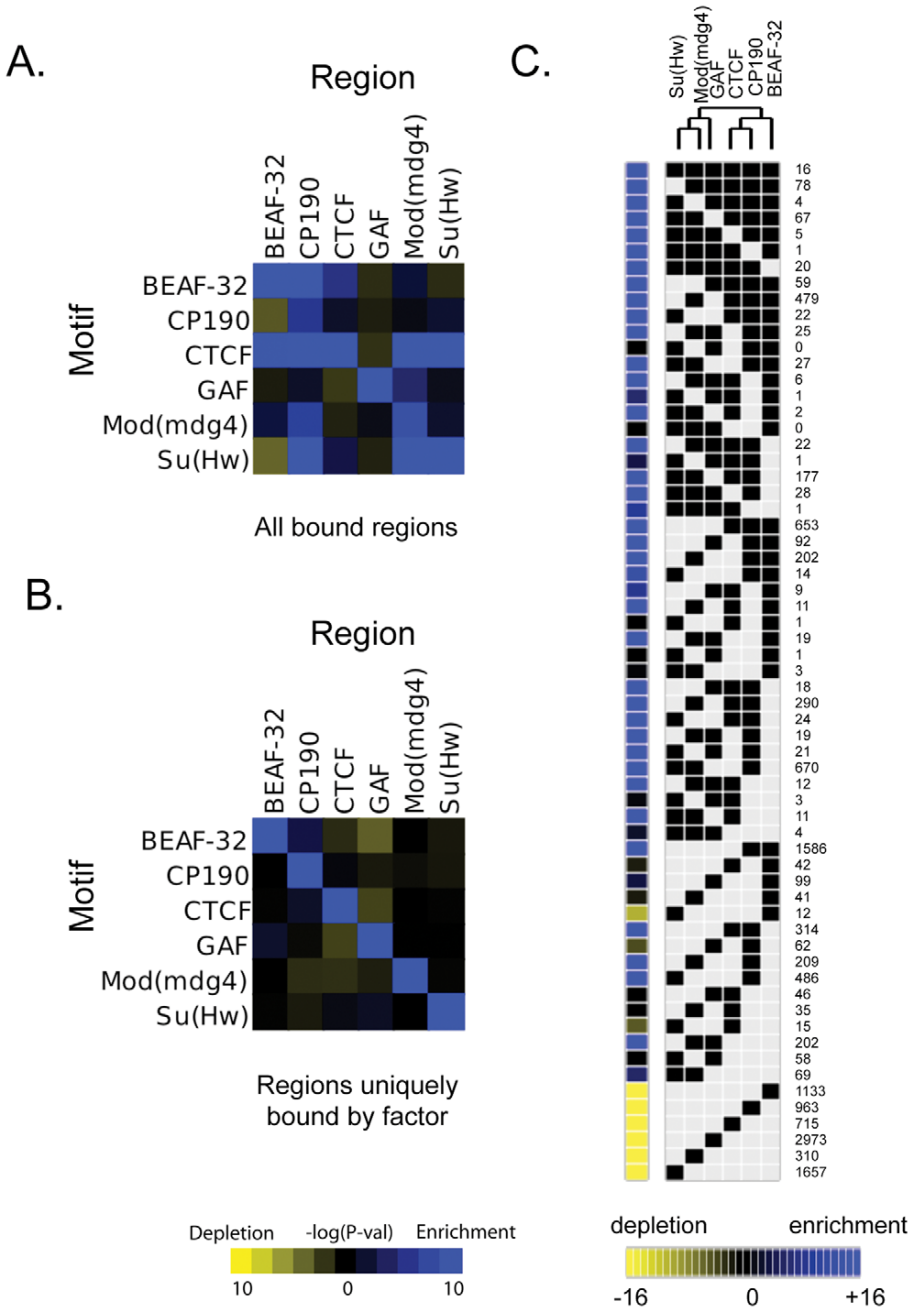
Combinatorial protein binding on insulators. For each insulator, intergenic bound regions were determined using the peaks ±100 bp (as described in Motif Discovery methods). The enrichment or depletion of instances of each motif with respect to these peak regions for each insulator is determined using a hypergeometric p-value as compared to control instances at 0.0 confidence, using (A) all bound regions for a particular protein or (B) peaks whose center is at least 1 kb away from the peak of any other insulator (uniquely bound regions). (C) All binding sites for all 6 insulator proteins have been classified based on their protein composition. Each of the categories is represented in the matrix as a black square for the factors associated with the binding site. The number of occurrences of each particular combination is indicated at the right of the matrix. The yellow to blue squares at the left of the matrix represent the enrichment or depletion p-value for each category when compared to simulated data. This matrix and the significant associations of factors have been use to build the dendrogram at the top of the figure.

To characterize the sequence specificity of each insulator-associated protein, we identified significantly enriched sequence motifs for each set of target sites (Figure S4). The most enriched motif identified for CTCF strongly resembles the CTCF motif identified in vertebrates [31] and *Drosophila* [10]. Likewise, the motif for Su(Hw) that was discovered in this study is similar to a motif previously identified in *Drosophila* from a limited number of Su(Hw) sites [14],[32],[33]. The discovered motifs are present in 75.6% of CTCF, 86.8% of BEAF-32, 84% of Su(Hw) and 88.6% of GAF binding sites (Table S3). Additionally, the motifs identified for one insulator-factor were often also enriched at the binding sites of other insulator-factors (Figure 2A). This cross-enrichment was not observed however, when only binding sites associated with a single factor were considered (Figure 2B), suggesting that each factor retains unique DNA-level binding specificity but associates with other insulator proteins via clustered binding sites and/or protein-protein interactions.

### Cell-type specificity of CTCF binding

Previous analyses have suggested that, in human cells, insulator binding sites are remarkably conserved across cell types [27],[31],[34]. Given the large overlap between binding sites here identified in whole embryos and data previously produced in *Drosophila* S2 cells for CTCF and CP190 [30], we investigated this trend further. We performed ChIP-chip experiments for CTCF in S2 and Kc cells. Approximately 74–81% of CTCF binding sites identified independently in each cell type overlap (specifically, have a midpoint to midpoint distance less than 250 bases)(Figure S5 and Figure S6A). This observation is consistent with a recently published analysis of CTCF binding sites in S2 and Mbn2 cells [35], in which, by the same criteria, 77–86% of binding sites overlap.

However, given the technical differences in protocols for embryos and suspension cell culture and the loss of information inherent in a comparison of independently thresholded binding site calls, we regard this as a conservative estimate. Qualitative observation of binding profiles suggests that many putatively differential binding sites may result from the threshold applied and normalization issues (Figure S5 and Figure S6B). Indeed, we note that the IP signals at non-overlapping binding sites are, on average, four-fold greater than input background, while overlapping binding sites are six-fold greater. In an attempt to avoid such biases, we used a linear mixed model framework to build a binding site detection model that jointly analyzes the data from multiple cell types (see Text S1). This model identifies 2,784 CTCF binding sites, only 166 of which show significant cell type specificity (Figure S7). In summary, while most insulator sites identified in this study appear to be conserved across cell types, a small fraction appear to function in a regulated fashion.

### Binding-site clustering identifies two major categories of insulator sequences

While the six insulator associated proteins mapped in this study often bind independently, we find clusters of overlapping binding sites far more often than would be expected by chance, indicating insulator-associated proteins often bind jointly to the same sequence. Indeed, 45% of the 14,145 binding sites identified in this study are occupied by more than one insulator associated protein. For example, 77% of CTCF binding sites cluster with at least another factor (Figure 2 and Table S1). Analysis of binding site cluster types revealed several notable trends (Figure 2C and Figure S3). CP190 is frequently (5690 out of 6651 total sites) found to bind with additional factors, BEAF-32 being its most common partner (3329/6651). BEAF-32, CTCF, and CP190 cluster together (1378/8872), as do Mod(mdg4) and Su(Hw) (1101/5381), while GAF displays a significant lack of clustering with other insulator proteins (2973 single sites out of 3905 total sites). This binding site clustering and the functional data presented below suggest a previously underappreciated compositional complexity of insulator sequences but also clearly identifies two major classes of insulators: Class I principally representing binding sites for BEAF-32/CP190/CTCF and Class II representing Su(Hw)-associated binding sites.

### Positional classification of insulators

**Figure 3:**
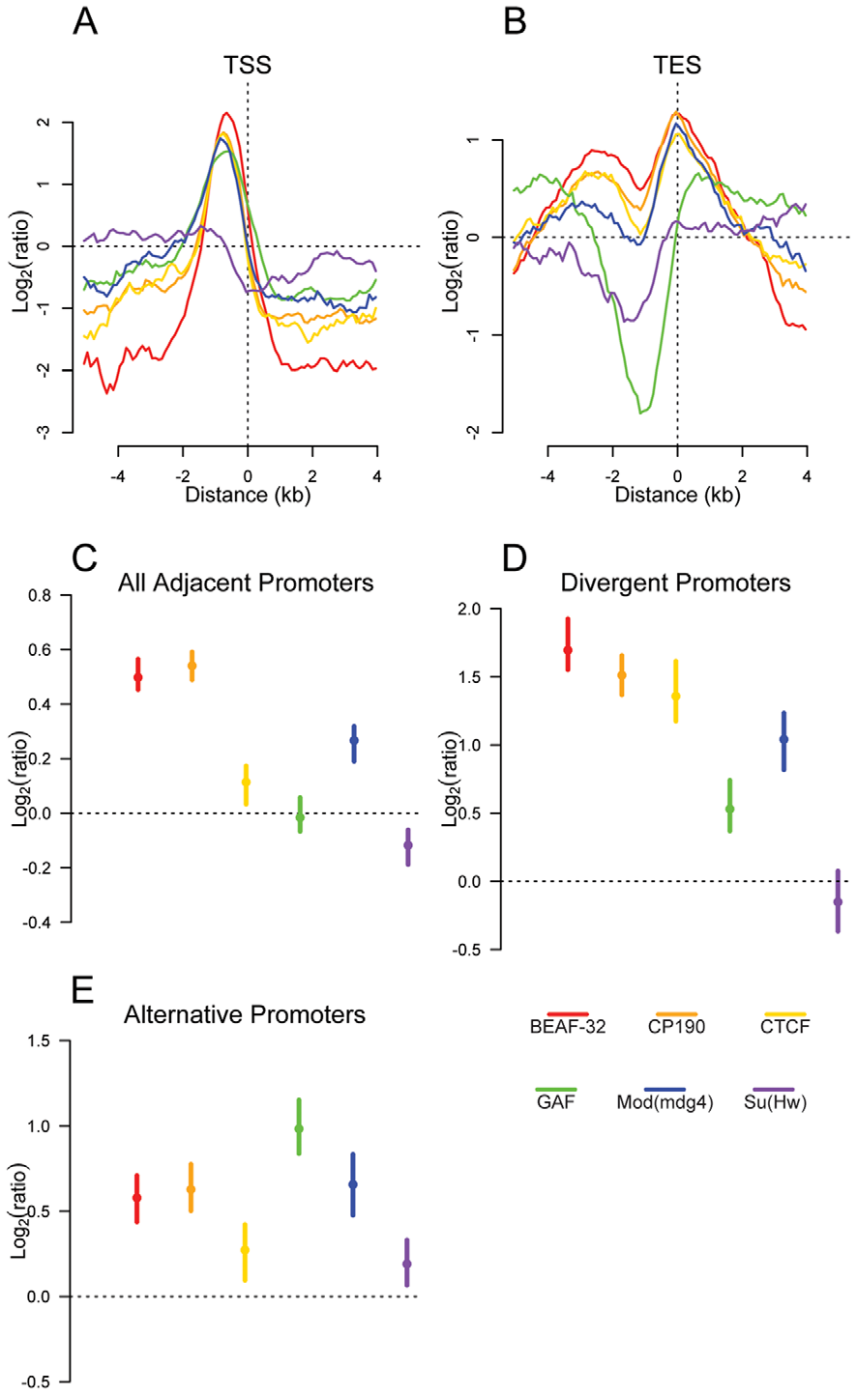
Insulator proteins are enriched between promoters. Plot of log_2_ enrichment or depletion of insulator binding sites (y-axis) by binding site base pair position (x-axis), relative to genomic transcript annotations. (A) Transcription start sites; negative and positive values depict upstream and downstream binding, respectively. (B) Transcription end sites; negative and positive values depict upstream and downstream binding. (C–E) Enrichment of insulator binding sites between adjacent promoters (C), divergent promoters (D), and alternative promoters (E). Points and lines depict enrichment estimates and 95% confidence intervals. A dotted line at 0 indicates no enrichment relative to the random expectation.

The distribution of insulator binding sites relative to different classes of functional genomic elements further supports the existence of several distinct functional classes of insulators. BEAF-32, CP190, CTCF, GAF, and Mod(mdg4) are clearly enriched at promoters (Figure 3A, Figure S8, and Table S4), while Su(Hw) is depleted. BEAF-32, CP190, CTCF and Mod(mdg4) binding sites are also strongly enriched within 5′UTRs as well as in intergenic regions (Figure S8) and at transcription end sites (Figure 3B). In contrast, they are largely excluded from transposable elements and coding exons (Figure S8 and Figure S9), suggesting a role of Class I insulator proteins, but not Class II, in regulating the transcription of genes.

We reasoned that if insulators act as gene boundaries, they should partition genes into distinct regulatory environments. Indeed, we find that four of the six insulator-associated proteins binding sites are significantly enriched between adjacent consecutive promoters (Figure 3C) with a stronger enrichment of BEAF-32, CP190, CTCF and Mod(mdg4) between adjacent divergently oriented promoters (Figure 3D). Additionally, as suggested previously in vertebrates for CTCF [31], Class I and Class II insulator proteins are significantly enriched between alternative promoters, providing a potential mechanism for their independent regulation (Figure 3E).

### Insulators demarcate differentially expressed genes

**Figure 4:**
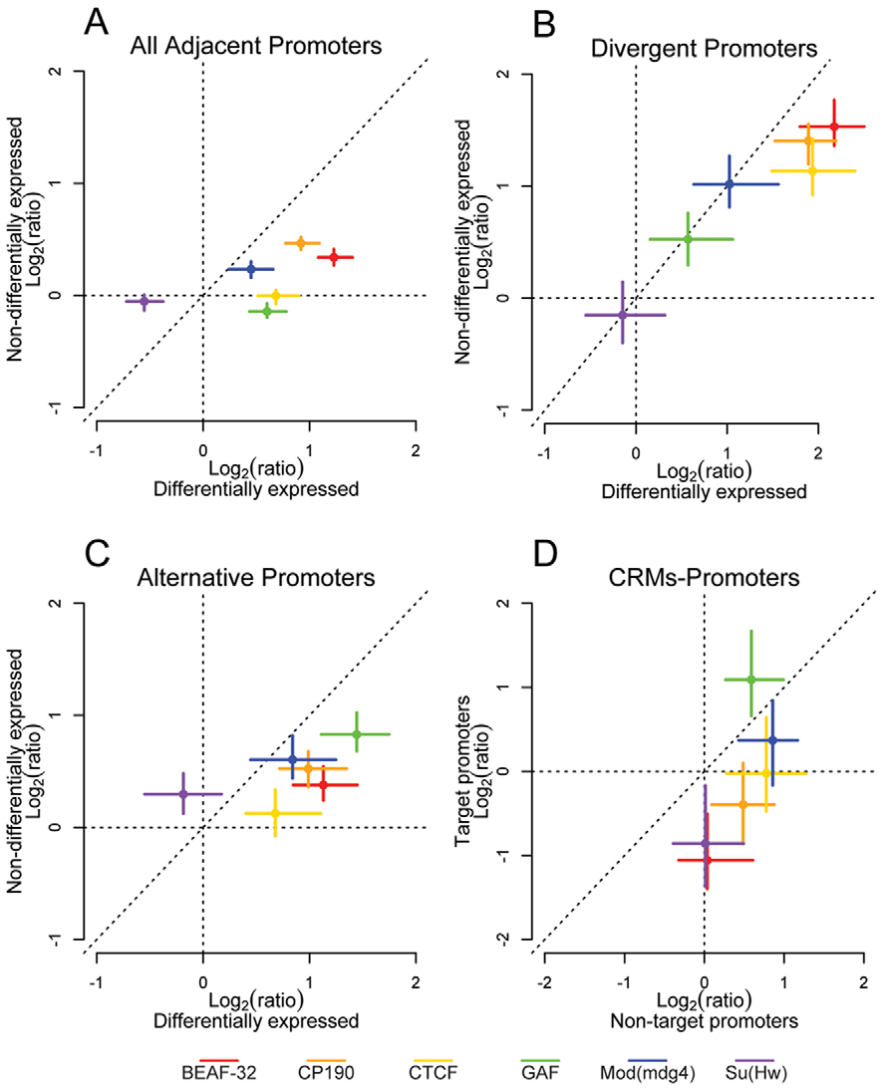
Insulator proteins segregate differentially expressed promoters. Log_2_ enrichment or depletion of insulator binding sites between (A) adjacent promoters, (B) divergently transcribed promoters, (C) alternative promoters, and (D) *cis*-regulatory elements and promoters. (A–C) X and Y axes depict enrichment between differentially and non-differentially expressed promoters, respectively. (D) X and Y axes depict enrichment between CRMs and their nearest non-target promoter and their target promoter. Points and lines depict enrichment estimates and 95% confidence intervals.

The distribution of insulators relative to a variety of genomic functional element classes suggests a pervasive role in controlling gene regulatory environments. To further address this hypothesis we mapped active promoters in embryos of the same developmental stage that we used for insulator mapping. To identify active promoters, we performed ChIP-chip with antibodies directed against the trimethylated lysine 4 of Histone H3 (H3K4me3), which is a clear mark of activation [36]–[39], and against the largest subunit of the RNA Polymerase II (PolII). We combined these two mappings with hybridization on tiling arrays of total RNA extracted from the same material. In *Drosophila* embryos, H3K4me3 is associated with gene Transcription Start Sites (TSS) and colocalizes with PolII immediately downstream of the TSS of active genes (Figure S10A and S10B). We extracted from this dataset a set of high confidence actively transcribed promoters, which overlap with H3K4me3 and PolII signals and whose exons overlap significant RNA signal (Figure S10C). We hypothesized that if insulators do indeed demarcate regulatory units, insulators would separate promoters with differing expression status. We repeated the positional analysis of insulator proteins between divergent, adjacent, and alternative promoters while taking into account the transcriptional status of the promoters (Figure 4A–4C). We observed that the enrichment of BEAF-32, CP190, CTCF, GAF, and Mod(mdg4) is greater between promoter pairs when they are differentially expressed (Figure 4, Figure S11, and Figure S12).

It is possible however that this result comes from an averaging of promoter activity across all the cell types present in the embryo at this developmental stage. We then repeated H3K4me3 ChIP-chip as a marker of active promoters in 2 embryonic *Drosophila* cell types: S2 and Kc cells. The overlap of H3K4me3 between embryos and Kc and S2 cell lines is between 71 and 75% respectively, while it is 85% between S2 and Kc cells (Figure S13A). Using H3K4me3 binding sites as a guide, we identified active promoters in each cell type. As in whole embryos, genes flanking CTCF binding sites identified in S2 and Kc cells show a significant enrichment of differentially expressed divergent and alternative promoters (Figure S13B and S13C) further demonstrating that Class I insulators delimit the boundaries of gene regulatory units.

### Insulators partition CRMs and promoters

Consistent with the limited previous functional data demonstrating the enhancer-blocking activity of insulators, we find binding sites for BEAF-32, CP190, and Su(Hw) are significantly depleted between annotated CRMs and their target promoters across the entire genome (Figure 4D, Figure S14), while CP190, CTCF, GAF, and Mod(mdg4) are enriched between *cis*-regulatory elements and their nearest non-target promoter, distributions that strongly support their proposed enhancer blocking function. Interestingly, we note that binding sites for GAF are significantly enriched between CRMs and their target promoters. Similarly, we find that BEAF-32, CP190, and Su(Hw) binding sites are depleted between distinct CRMs of the same gene, while GAF is found more frequently than expected (Figure S14).

We note that the enrichment of insulators within such genomic features may, in part, be driven by the effects of differential promoter density or biases in chromatin accessibility. In order to understand how such factors could affect any interpretation of our data, we reanalyzed binding site data for 36 recently published datasets corresponding to 21 transcription factors, from the Berkeley *Drosophila* Transcription Network Project (BDTNP) [40]. We first observed that none of our insulator binding sites preferentially localize with this transcription factor set (Figure S15). Despite several transcription factors that preferentially bind promoter-proximal sequences (Figure S16), the enrichment of insulators between promoter pairs is greater than for any of the published transcription factors (Figure S17A, S17B, S17C). In contrast to these findings, and as expected, the published BDTNP transcription factors are not as strongly biased towards CRM, non-target promoter separation (Figure S17D).

### Insulators mark the boundary of chromatin domains

**Figure 5:**
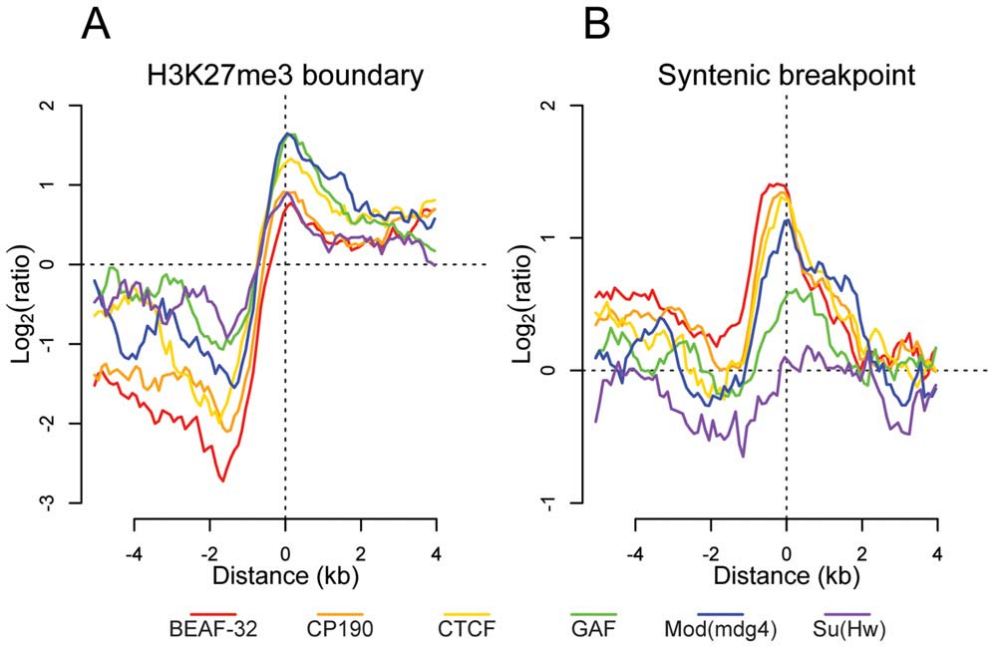
Insulator proteins mark chromatin and syntenic block boundaries. Log_2_ enrichment or depletion of insulator binding sites. (A) H3K27me3 boundaries; negative and positive values depict binding within and outside regions of histone modification. (B) Syntenic breakpoints; negative and positive values depict binding within and outside syntenic blocks.

Previous studies have demonstrated that insulators delimit distinct organizational domains of a genome [27],[30]. One such chromatin domain is marked by the trimethylated Lysine 27 of Histone H3 (H3K27me3), a histone modification deposited and recognized by the repressive Polycomb protein complexes [41]. We mapped by ChIP-chip the H3K27me3 mark in *Drosophila* embryos. We observed in whole embryos, as described previously [42],[43], that H3K27me3 is distributed throughout the genome in large domains (Figure S18). To better define the boundaries of these large genomic regions, we used a hidden Markov model based segmentation algorithm. We confirm that the genes affected by this silencing mark correspond to the previously described Polycomb target genes [42]–[45]. We identified 140 regions of substantial H3K27me3 density and quantified the distribution of each insulator binding site type with respect to the domain boundaries. Interestingly we find that all 6 factors are significantly depleted within and enriched outside these regions (Figure 5A). In addition, CTCF, GAF, and Mod(mdg4) are enriched at the boundaries of regions of high H3K27me3 density, with this enrichment significantly decreasing at increasing distances, further supporting the insulators' role in chromatin domain boundary determination (Figure 5A and Figure S18). It is possible that this result is confounded by the fact that insulators are enriched at TSSs. We performed Pearson's chi-squared contingency table tests to assess if the frequency of insulator-H3K27me3 boundary overlaps are independent of (and greater than) the frequency of TSS- H3K27me3 boundary overlaps. Indeed, CP190 (p<9.8e-6), BEAF-32 (p<1.8e-5), CTCF (p<0.00013), GAF (p<0.0022), Mod(mdg4) (p<0.00035), and Su(Hw) (p<0.0088) are independently associated with H3K27me3 breakpoints.

### Insulator binding site sequence constraint and conservation of synteny

Given their apparently pervasive role in the establishment of gene regulatory units, we examined the role insulator sequences have played in shaping the evolution of the *Drosophila* genome. First, insulators show evidence of local sequence constraint. Based on either 15-way insect multiple sequence alignments or pair-wise alignments between the closely related *Drosophila melanogaster* and *Drosophila simulans*, insulator binding sites evolve significantly slower than fast evolving introns, although more swiftly than either coding exons or most transcription factor binding sites [46] (Figure S19). Second, we find that BEAF-32, CP190, CTCF, and Mod(mdg4) are significantly enriched near the 12 *Drosophila* species syntenic breakpoints (Figure 5B) [47]. Chi-squared tests demonstrate that for CP190 (p<0.0031), BEAF-32 (p<0.0086), GAF (p<0.027), and Mod(mdg4) (p<0.034), this result is independent of the association of TSSs and syntenic breaks. This finding provides evidence to support the hypothesis [48] that selective pressure has maintained gene regulatory units established by flanking insulators.

### Insulators are sites of dynamic chromatin

**Figure 6:**
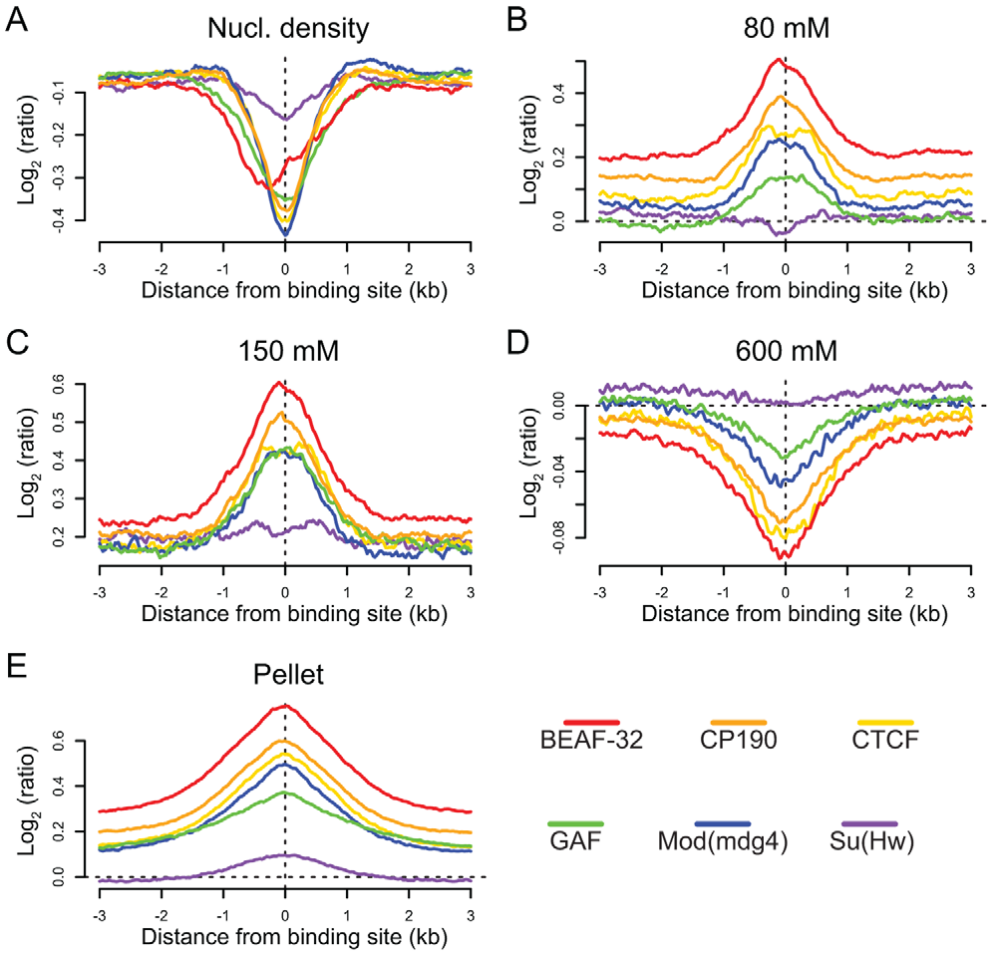
Insulators are sites of dynamic chromatin. Nucleosome density and salt fractionation profiles for *Drosophila* S2 cell chromatin, aligned at sites of insulator protein binding as indicated by color and averaged over a ±3 kb region. (A) nucleosome density, (B) 80 mM salt fraction, (C) 150 mM salt fraction (D) 600 mM salt fraction, (E) salt-washed pellet.

We find that binding sites for 5 of the 6 insulator-associated proteins (Su(Hw)is the exception) are regions of reduced nucleosome density relative to surrounding regions (Figure 6A). Reduced nucleosome density often corresponds to sites of high histone replacement or displacement [49],[50] and classical “active” chromatin as defined by salt solubility properties [51]. We also find that the same 5 of the 6 insulator proteins are preferentially bound in regions characterized by low-salt soluble nucleosomes (Figure 6B and 6C), depleted in the remaining high-salt-soluble fraction (Figure 6D) and highly enriched in the salt-washed insoluble pellet (Figure 6E). Similar analyses of only non-promoter proximal insulators reveal the same trends, indicating that the shared solubility properties of insulators and promoters are indeed independent (Figure S20). Given the correspondence between these results and the regulatory boundary analyses presented above, we hypothesize that this difference in chromatin properties may explain why Su(Hw), defining ClassII insulators, does not act as a gene boundary in the genome.

## Discussion

Insulator identification has been the source of much recent interest. Indeed, in the last 6 months CTCF was mapped in S2 cells [52]; BEAF-32 in embryos (6–16 h of development) [53], CTCF and CP190 in S2 cells [54] and more recently CTCF, Su(Hw), CP190 and BEAF-32 in Kc cells and Mbn2 cells [35]. Interestingly, the latter paper describes three subclasses of insulators, with CP190/BEAF association being distinct from CP190/CTCF and CP190/Su(Hw).

We present in this study the embryonic binding profile of six factors previously known to be associated with insulator function in *Drosophila*. Our analysis of insulator binding site distributions and protein composition suggest there exist 2 principal categories of insulator elements (Class I and Class II). In particular, we have shown that Class I insulators, identified by the binding of CTCF, CP190 or BEAF-32, segregate differentially expressed genes and delimit the boundaries of chromatin silencing, while they are depleted between known CRMs and their target genes. We do not find evidence supporting a significant distinction between CP190/BEAF and CP190/CTCF or CTCF/BEAF. In contrast, our analyses suggest that BEAF-32, CP190, and CTCF are distributed and function quite similarly, while Su(Hw) appears distinct. The Class II insulators, bound by Su(Hw), are often exceptional in our analyses. We note that the analysis of genome-wide mapping data, expression data, and genome annotation provides an endogenous boundary assay that demonstrates that, while Su(Hw) has been described as an insulator before, it is not systematically associated with the boundaries of the gene units.

**Figure 7:**
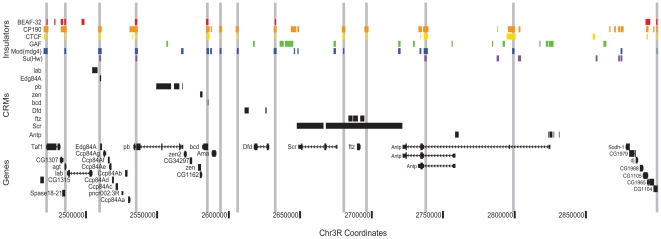
Class I insulators demarcate regulatory boundaries in the Antennapedia Complex (ANT-C) region. Binding sites for insulator proteins are depicted as colored boxes at top. For display purposes, grey vertical lines are drawn through all positions bound by two or more class I insulators. ORegAnno (www.oreganno.org) defined *cis*-regulatory modules for each of 9 genes are plotted as black boxes across the middle. RefSeq (www.ncbi.nlm.nih.gov/RefSeq/) gene models and coordinates across the 500 kb Antp region are displayed at bottom.

By helping to delimit the regulatory boundaries of genes, the Class I insulator map presented here will aid in the identification of transcription factor target genes and the construction of transcriptional regulatory networks. As an example of this concept, we illustrate the distribution of known regulatory elements and insulators across the Antennapedia Complex (ANT-C) of homeotic genes (Figure 7). This region quite strikingly demonstrates the potential utility of insulator binding data for *cis*-regulatory annotation. Across approximately 500 kb, *cis*-regulatory elements and their target promoters are found between insulator pairs. For example, a single insulator separates the *lab* and *Edg84A* genes, with their respective *cis*-regulatory elements narrowly partitioned on either side. The adjacent regulatory elements and promoters of *zen* and *bcd* are similarly insulator segregated.

The presence of an insulator 3′ of *ftz* was previously hypothesized [55] to explain the ability of distal *Scr* regulatory elements to bypass *ftz* by pairing with the proximal SF1 insulator, located between *Scr* and *ftz*. Lastly, at *Antp*, as we observe genome wide, two alternative promoters and their proximal regulatory elements are segregated by a single insulator. We are currently developing analysis methods to systematically partition the entire genome into such regulatory domains.

Consistent with their observed regulatory boundary functions, Class I insulators are embedded within local regions of active chromatin and are frequently associated with syntenic breakpoints between species. Previous work has demonstrated that active promoters in yeast and *Drosophila* are associated with reduced nucleosome occupancy and low-salt soluble and high-salt insoluble chromatin [50],[56] (Figure S20). Therefore, surprisingly, dynamic chromatin is a shared feature between promoters and most classes of insulators. It is notable however that some studies have revealed functional similarities between insulators and promoters in transgenic assays [57]. These results have been described as paradoxical, as insulators can negatively affect promoters by blocking communication between enhancers and promoters. One proposed model for insulator function is that they act as promoter “decoys” by recruiting away factors necessary for transcriptional initiation [57]. Alternatively, insulators and promoters might require common chromatin features to function by mechanisms that are still unknown. One potential interpretation is that the dynamic chromatin at insulators forms a flexible chromatin joint that would affect the probability of productive contact between separated regulatory elements. In this way, the similarity between promoters and insulators would be a consequence of their common requirement for dynamic chromatin, although with very different consequences. This model may explain why promoters are so frequently scored as insulators in the classical insulator assay, when an element is placed between an enhancer and a promoter [1],[58].

## Materials and Methods

### ChIP–chip

Chromatin immunoprecipitations have been performed as described previously [59]. Briefly, the biological material is homogenized in the presence of 1.8% formaldehyde. The cross-linked chromatin is sonicated using a Bioruptor (Diagenode) to an average size of 500 bp. Pre-cleared chromatin extract is incubated overnight at 4C with the specific antibody and immunoprecipitated with protein-A Sepharose beads. After purification of the DNA and amplification of the libraries by linker-mediated PCR, the samples are labeled according to Affymetrix protocols and hybridized in parallel with an input sample onto the Affymetrix *Drosophila* Tiling Array, v2.0 R.

### Antibodies

CTCF-C and CTCF-N antibodies are described in [8], CP190 antibody is described in [60], BEAF-32 antibody is described in [18], Mod(mdg4) antibody is directed against the 67.2 isoform and is described in [61], Su(Hw)-1 antibody is described in [62], Su(Hw)-2 is described in [63], GAF antibody is described in [64], H3K27me3 antibody is from Upstate (07-449 lot DAM1387952), H3K4me3 antibodies is from Abcam (ab8580 lot 411277) and PolII antibody is from Covance (8wG16 lot 14861301).

### Analysis of arrays

Insulator binding data was processed with Model based Analysis Tiling-arrays (MAT) software [65]. We ran paired MAT analysis with MaxGap of 500, MinProbe of 10, and a Bandwidth of 250. H3K4me3, PolII and RNA data were analyzed with TAS (Tiling Array Software) and a threshold of 5% of the highest pValues was applied to identify the high intensity signals. The same parameters as for the MAT analysis have been applied to then call the peaks with TAS.

We developed a new HMM-based segmentation algorithm to identify H3K27me3 domains, as well as a novel mixed model framework for the joint analysis of ChIP-chip data from more complicated experimental designs, here applied to CTCF binding data from multiple cell types (see details in Text S1).

### Motif discovery

Motif discovery was performed separately for each insulator. Peak centers that were at least 1 kb away from the peak center of any other insulator were taken (“uniquely bound peaks”) and +/− 100 bp windows were generated excluding coding exons, repeats, transposons, 3′ untranslated regions and non-coding RNAs (“excluded regions”). For each insulator up to 500 of the regions were randomly selected and enriched motifs were identified using MEME [66], AlignACE [67], and MDscan [68]. All programs were run with default parameters except for MEME, which was restricted to a maximum of 3 iterations and a maximum motif width of 25. Instances of each of the motifs at conservation levels from 0.0 to 1.0 confidence (in steps of 0.1) were identified in all Intergenic regions (defined as genomic regions excluding those noted above) using the motif instance pipeline described in [69] with a PWM threshold corresponding to a p-value of 4^−8^ as determined by TFM-Pvalue [70]. The motifs were ranked using the fraction of instances found in the uniquely bound regions divided by the fraction for instances of shuffled control motifs at the same conservation cutoff (Wilson's confidence interval at Z = 1.5 was used on the ratios to give a conservative enrichment). This procedure is designed to reduce biases due to composition or conservation level. The motif with the highest enrichment at any confidence level was selected. This procedure was repeated using the MAT peak regions (rather than +/− 100 bp) to produce the comparison in Figure S4 (otherwise the +/− 100 bp motifs are used throughout).

### Genomic distribution analyses

Genomic distribution analyses only used insulators mapped to chromosomes 2L, 2R, 3L, 3R, 4, and X. All gene annotations, including transcription start site locations and alternative promoter presence were defined according to RefSeq annotations. Transposable element locations were based on Flybase annotations. Divergently transcribed genes were identified as all adjacent transcription start sites, on opposite strands, between 500 and 2500 bases apart. Alternative promoters were identified as all RefSeq annotated genes with more than one distinct transcription start site. The ‘all adjacent’ gene set included all adjacent gene pairs whose transcription start sites were between 1500 and 20000 bases apart, regardless of strand. *Cis*-regulatory elements and their target genes were defined according to the RedFly database [71]. Breakpoints of regions of conserved synteny across the 12 sequenced Drosophilids were identified in [47].

All genomic distributional analyses were first conducted by mapping protein binding sites relative to the genomic feature of interest. This mapping was performed in one of two ways; First, for genomic features that can be faithfully represented as a single base (e.g., a transcription start site), the distance from each insulator to its nearest feature was tabulated, second, for paired genomic features (e.g., divergent promoters), the number of intervening insulators for each feature pair was tabulated. To quantify if the distribution of mapped insulators relative to the genomic feature of interest is significantly different than would be expected by chance (given the number of insulators and the distribution of the particular feature of interest), we performed simulations as follows. First, permuted insulator binding sites were generated by sampling n sites from a random, uniform distribution, the length of each chromosome, where n is the number of observed insulator binding sites, by chromosome. In other words, a simulated insulator is equally likely to be placed at any location across a chromosome. Second, the simulated binding sites were mapped relative to the genomic feature of interest, as with each real dataset. This procedure was repeated 10,000 times for each insulator, target element combination. The median simulated values were used to normalize the real data counts to produce enrichment estimates. The 2.5 and 97.5 percentiles of the simulated distributions were used to produce confidence intervals for display purposes and significance estimates. Empirical p-values were calculated as the fraction of simulations that produced a number of mapped features as extreme as observed in the real data.

### Nucleosome enrichment and salt fractionation

The position of binding sites have been compared to data of nucleosome density and salt fractionation of the chromatin extraction as described in [51]. Binding sites are defined by their midpoint and nucleosome density and salt fractionation data from S2 cells are plotted as a log ratio of enrichment in a 3 kb interval around the midpoint of the binding site.

### GEO accession number of described datasets

GSE16245

## Supporting Information

Figure S1 Example of mapping around some known insulators. The vertical dotted line indicates the location of the known insulators: (A) the 1A2 insulator [16],[17] in the *yellow* locus, (B) the scs and scs' elements [18] in the hsp70 locus, (C) the SF1 insulator in the ANT-C region [19].
(0.92 MB JPG)

Figure S2 Pair-wise overlap at varying distance thresholds. In this example, the overlap between peaks at 1% FDR for CTCF-N and each of the other factors is plotted. The y axis represents the number of overlapping binding sites, while the x-axis represents the minimal distance between two peaks to call them overlapping. The plateau between CTCF-N and CTCF-C, which correspond to two independent antibodies for CTCF, is reached at a distance of 250 bp, which is the minimal distance we used for further analyses.
(0.31 MB JPG)

Figure S3 Overall pair-wise comparison between different factors. The axes in the radar plots indicate the percentage of overlapping binding site for one factor compared to each of the other factors. Data for CTCF and Su(Hw) corresponds to the CTCF_C and Su(Hw)-1 datasets respectively. This representation allows a quick identification of the preference of association between factors. For example, GAF is principally associated with itself and no other factor, while CTCF overlaps to a greater extent with CP190, Mod(mdg4), and BEAF-32, but not with GAF and Su(Hw).
(0.53 MB JPG)

Figure S4 *de novo* Identification of DNA motifs. The newly discovered motifs for each factor are represented in color logos, while the previously known motifs are represented in gray scale. We present the motifs corresponding to 2 different discovery regions: the original peak regions as called by MAT (noted Binding Regions; median size ∼1,000 bp) and ±100 bp around the center of each peak (see Materials and Methods). The newly discovered motifs for CTCF, Su(Hw) and GAF are in agreement with previously described motifs [8],[10],[20], while the motif discovered for BEAF only agrees with previous studies [21],[22] when discovery is performed using the smaller ±100 bp regions. Interestingly, using the larger MAT regions, high information content motifs are identified for both CP190 and Mod(mdg4) which are not thought to bind DNA directly. The CP190 motif matches a known Vertebrate centromeric sequence [23]. However, the top motifs discovered using the ±100 bp regions are highly degenerate suggesting that while the factors may not bind the DNA directly, co-factors might bind in the more distant vicinity of their peaks.
(0.75 MB JPG)

Figure S5 CTCF is a constitutive feature of the *Drosophila* genome. (A,B) In these genome browser views the ChIP-chip profiles for CTCF-C and CTCF-N in embryos are represented as top two tracks. Also represented are the ChIP-chip profiles for CTCF-N in two different cell lines: S2 cells and Kc cells.
(0.62 MB JPG)

Figure S6 Decreased signal intensity at cell-type specific CTCF binding sites. (A) A Venn diagram showing the overlap between the binding sites for CTCF in embryos, in S2 cells and Kc cells. (B) The mean and standard deviation of the fold change for each pair-wise comparison between CTCF-C [embryos] and CTCF-N [embryos, S2 cells, Kc cells] is plotted for the peaks that do overlap, and the peaks that don't. The same statistical criteria applied to different datasets might not represent the variation between the different biological samples.
(0.39 MB JPG)

Figure S7 A joint-model analysis of the binding sites of CTCF in different tissues. All the raw data from CTCF ChIP-chip in different tissues have been analysed together with a joint model (see Text S1). A p value corresponding to 1% FDR has been applied to identify the binding sites. The same p value threshold has been applied to estimate the statistical difference of a peak in one condition compared to the others. (A,B) A comparative genome browser view of the results obtained by the joint model and a MAT analysis. In the first example (A) no difference is detected among the 3 profiles, while in (B) a binding site for CTCF upstream of the Fas3 gene is absent in Kc cells.
(0.65 MB JPG)

Figure S8 Distribution of the different classes of insulator binding sites compared to genomic features of *Drosophila*. (A) Barchart indicating the number of insulator binding sites of each class mapping to 5′ UTRs (red), exons (blue), introns (green), 3′ UTRs (purple), and intergenic regions (orange). For comparison, this distribution is also plotted for the set of transcription factors from MacArthur et al. [24] and for H3K4me3. (B) Data as in (A) normalized within each class to illustrate the fraction of insulators mapping to each annotation type. Also plotted at the right of the graph is the percentage of each region present in the Dm3 assembly of the *Drosophila* genome.
(0.57 MB JPG)

Figure S9 Distribution of the distance of insulator proteins binding sites relative to Transposable Elements. Estimated enrichment of insulator binding sites (black lines), with flanking 95% confidence intervals (gray lines) (Y-axis) are plotted against binding site base pair position (x-axis), relative to transposable element boundaries. Negative positions indicate binding sites within an annotated transposable element, 0 indicates the element boundary, and positive values represent positions outside and flanking element annotations.
(0.53 MB JPG)

Figure S10 Expression status of *Drosophila* embryos. (A,B) Enrichment and 95% confidence intervals (Y-axis) plotted against distance to transcription start sites (x-axis) for identified PolII enriched regions (A) or H3K4Me3 enriched regions (B). (C) Venn Diagram representing genes associated with a PolII binding sites at their TSS, an H3K4me3 mark at their TSS and a RNA signal on their exon.
(0.34 MB JPG)

Figure S11 Example of position of insulator binding sites at divergent promoters. A genome browser example of signal obtained by ChIP-chip for H3K4me3 (purple), PolII (red), as well as total RNA profiling on tiling microarrays (orange). Insulator binding sites are also represented in this example where we can observe that a Class I insulator, defined by the binding of CTCF, CP190, BEAF-32, and Mod(mdg4), is located between the divergent genes CG6509 and CG4970 which are separated by approximately 350 bp. CG6509 is transcribed as identified by its RNA level and have an active promoter, as identified by the presence of PolII and H3K4me3 at its TSS. CG4970, however, is inactive, thus suggesting that the presence of the insulator allows CG4970 to be activated independently of CG6509.
(0.31 MB JPG)

Figure S12 Distribution of insulator binding sites around the TSS of genes dependent of their transcription status. Log enrichment or depletion of insulator binding sites (y-axis) are plotted against binding site base pair position (x-axis), relative to the transcription start sites; negative and positive values depict upstream and downstream binding, respectively. Each panel corresponds to cases where the promoter is either active (On) or inactive (Off), as defined by the presence or absence of H3K4me3 and PolII (Figure S10C) and the transcriptional status of the nearest upstream promoter. (A) The gene TSS is on and the nearest upstream TSS is on. (B) The gene TSS is On and the nearest upstream TSS is Off. (C) The gene TSS is Off and the nearest upstream TSS is Off. (D) The gene TSS is Off and the nearest upstream promoter is On.
(0.50 MB JPG)

Figure S13 Enrichment of CTCF binding sites between Adjacent Promoters in different cell lines. (A) Venn diagram between H3K4me3 associated promoters in embryos, S2 cells and Kc cells. Enrichment and 95% confidence intervals of CTCF binding sites in embryos, S2, and Kc cells between (B) divergent and (C) alternative promoters.
(0.34 MB JPG)

Figure S14 Enrichment of different classes of binding sites between CRMs and Promoters. For each insulator binding site class, enrichment estimates and flanking confidence intervals (Y-axis) are plotted for genomic intervals with 0, 1, or 2 insulator binding sites (X-axis). Intervals are defined by the region between *cis*-regulatory elements and their target promoters (red), or between *cis*-regulatory elements and their nearest non-target promoters (black), or between adjacent *cis*-regulatory elements that regulate the same gene (gray).
(0.36 MB JPG)

Figure S15 Insulator proteins do not associate with transcription factors binding sites. Binding sites from 36 datasets corresponding to 21 transcription factors [24] were downloaded from UCSC and compared to our set of insulator binding sites. A distance matrix was constructed as 1 minus the fraction of sites with midpoint to midpoint distances less than 250 bases (data in Table S4) and hierarchically clustered using the average linkage method. Cell colors range from blue to white to red to depict increasing site overlap.
(0.59 MB JPG)

Figure S16 Enrichment at the TSS of insulator proteins and transcription factors. Binding sites from 36 datasets corresponding to 21 transcription factors (BDTNP; [24]) were downloaded from UCSC and compared to our set of insulator binding sites. Log2 enrichment or depletion of insulator binding sites and associated 95% confidence intervals (Y-axis) are plotted against binding site base pair position (X-axis), relative to the transcription start sites; negative and positive values depict upstream and downstream binding, respectively. A point of comparison for a promoter associated factor TFIIB is represented in light blue.
(0.59 MB JPG)

Figure S17 Promoter demarcation by insulators and transcription factors. Same Legend as in Figure 4. Now represented in black are the data corresponding to the BDTNP datasets. TFIIB is represented in light blue.
(0.53 MB JPG)

Figure S18 CTCF at the boundaries of H3K27me3 domains. Genome browser example showing signals for CTCF and H3K27me3 ChIP-chip experiments performed in embryos and S2 cells. The H3K27me3 data in S2 cells is reanalyzed from [3] (see Text S1). HMM segmentation is used to define the boundaries of H3K27me3 better. This can be visualized on these examples when compared with a MAT analysis performed on embryos. The dashed vertical lines show CTCF binding sites at domain boundaries. (A) Example of CTCF bordering an H3K27me3 domain covering the btd/Sp1 locus. (B) In this example, an entire H3K27me3 domain corresponding to the *Abd-B* gene disappears, while this chromatin mark is maintained in the rest of the Bithorax Complex region. This depletion of the H3K27me3 mark corresponds to *Abd-B* being expressed in S2 cells while *Ubx* and *abd-A* are repressed. Embryos corresponding to a mixed population of cells, the H3K27me3 signal is coming from its presence in a subpopulation of cells. All CTCF binding sites in this region are conserved between S2 cells and embryos, even inside the H3K27me3 depleted domain (between the dashed vertical lines) and are therefore independent of the transcriptional status of *Abd-B*. Furthermore, the breakpoints of the H3K27me3 depleted domain in S2 cells, compared to embryos, correspond to CTCF binding sites (represented by the two vertical dashed lines).
(0.78 MB JPG)

Figure S19 Conservation of insulator binding sites. Phastcons between 15 insect species, including the 12 sequenced *Drosophilae* species, have been calculated for each category of insulator binding sites. The bars correspond to the median (dot) and median absolute deviation (bars) of the scores. The dark red bar (FEI) corresponds to the same scores calculated for fast evolving introns (neutral reference). Also plotted for reference are exons (pink), H3K4me3 (gray), and BDTNP binding sites (black).
(0.30 MB JPG)

Figure S20 Dynamic chromatin at insulator binding sites at non-promoter and promoter sites. Each insulator site, defined as the midpoint of the binding site interval, was classified as to whether it fell within the interval defined by a transcriptional start site and 500-bp upstream, using the 12,807 unique 5′ ends annotated in FlyBase r5.13. Based on these criteria, the number of sites in non-promoters and promoters are: BEAF-32 (5546 nonpromoters, 2281 promoters); CP190 (7758 non-promoters, 2698 promoters); CTCF (3286 non-promoters, 1146 promoters); Mod(mdg4) (3154 non-promoters, 821 promoters); GAF (5551 non-promoters, 887 promoters); Su(Hw) 4565 non-promoters, 214 promoters). Displays are for non-promoters (A-E) and promoters (F-J) using the same datasets shown in Figure 5. (A,F) Nucleosome density; (B,G) 80 mM salt fraction; (C,H) 150 mM salt fraction; (D,I) 600 mM salt fraction; (E,J) salt-washed pellet.
(0.82 MB JPG)

Table S1 Binding sites of the insulator-associated proteins. The number of binding sites per factor at different confidence interval generated by MAT analysis.
(0.02 MB XLS)

Table S2 Known insulators detected by our ChIP-chip analysis. For each published functional insulator element, an X mark indicates if they are associated with a peak for each factor identified in our ChIP-Chip experiments.
(0.02 MB XLS)

Table S3 Binding sites containing a discovered consensus motif. Number of regions containing the corresponding discovered motif (see Motif Discovery methods) for each factor at different PWM thresholds (e.g. 6 indicates matching the genome with 4^−6^ p-value and is the most lenient threshold). The 4 panels represent the set of regions studied (all regions versus uniquely bound regions) at different scanning windows (±100 bp and ±1000 bp around the peak centers). The numbers in each cell indicate the number of intergenic peaks that contain a motif/the total number of intergenic regions. The number after each colon indicates the enrichment of motif instances inside the considered regions (compared to the fraction of the intergenic genome the regions represent). Motifs are in general good predictors for CTCF, Su(Hw), BEAF-32, and GAF (as evidenced by the high enrichment). Within a distance of ±1,000 bp, and at a PWM p-value of 4^−6^, the discovered motifs are present in 75.6% of CTCF, 86.8% of BEAF-32, 84% of Su(Hw) and 88.6% of GAF binding sites.
(0.03 MB XLS)

Table S4 Binding sites overlaps. Fraction of each factor's binding sites with midpoint to midpoint distances less than 250 bases, for each other factor type studied.
(0.07 MB XLS)

Text S1 Supplementary methods.
(0.07 MB DOC)
