# Supplementary figures and images for: A Comprehensive Map of Insulator Elements for the Drosophila Genome
Source: PLoS Genet. 2010 Jan 15;6(1):e1000814. doi: 10.1371/journal.pgen.1000814 (PMC2797089; doi:10.1371/journal.pgen.1000814)

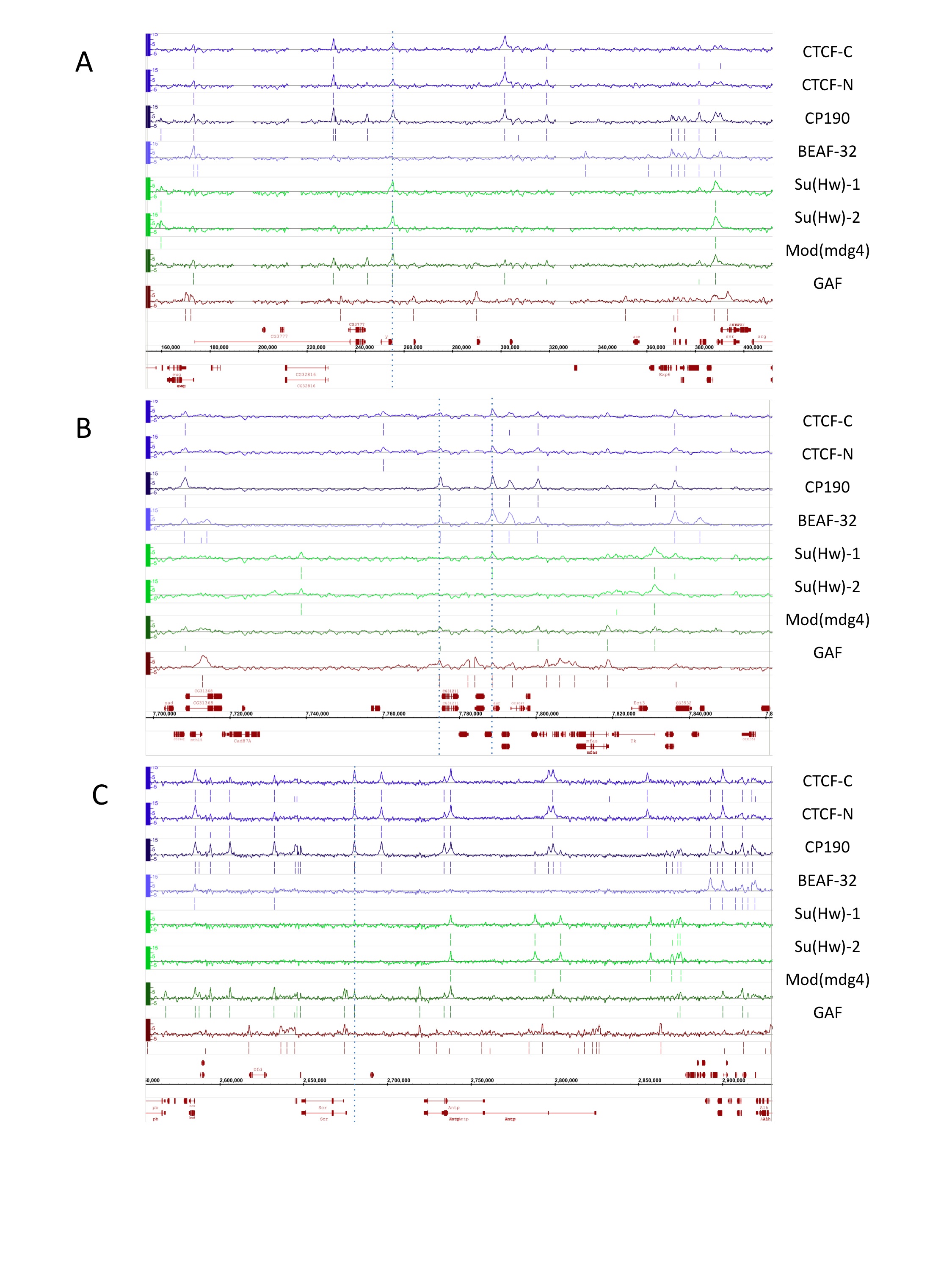

Supplement: Figure S1 — Example of mapping around some known insulators. The vertical dotted line indicates the location of the known insulators: (A) the 1A2 insulator [16],[17] in the yellow locus, (B) the scs and scs' elements [18] in the hsp70 locus, (C) the SF1 insulator in the ANT-C region [19]. (0.92 MB JPG) [file pgen.1000814.s001.jpg]

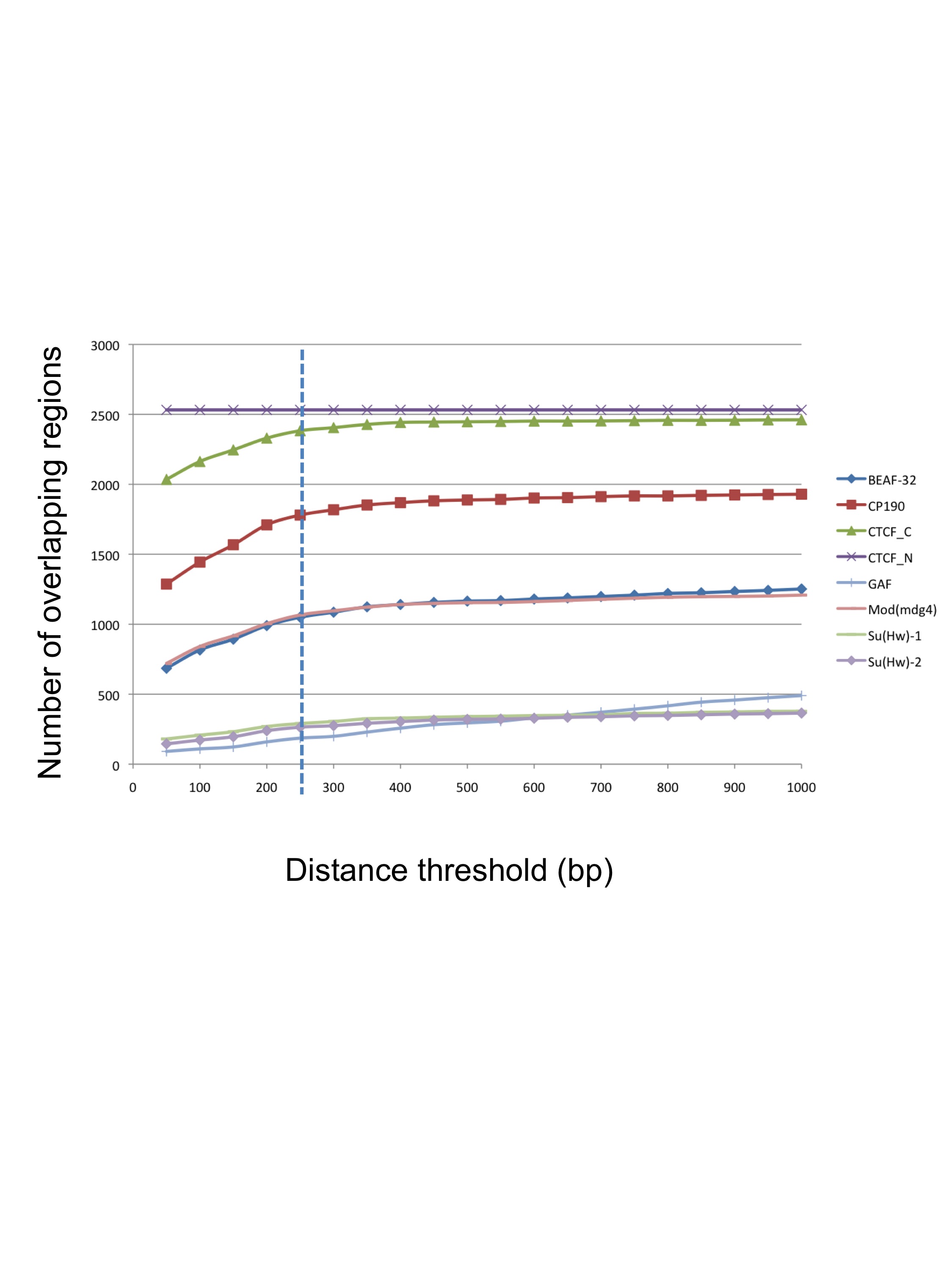

Supplement: Figure S2 — Pair-wise overlap at varying distance thresholds. In this example, the overlap between peaks at 1% FDR for CTCF-N and each of the other factors is plotted. The y axis represents the number of overlapping binding sites, while the x-axis represents the minimal distance between two peaks to call them overlapping. The plateau between CTCF-N and CTCF-C, which correspond to two independent antibodies for CTCF, is reached at a distance of 250 bp, which is the minimal distance we used for further analyses. (0.31 MB JPG) [file pgen.1000814.s002.jpg]

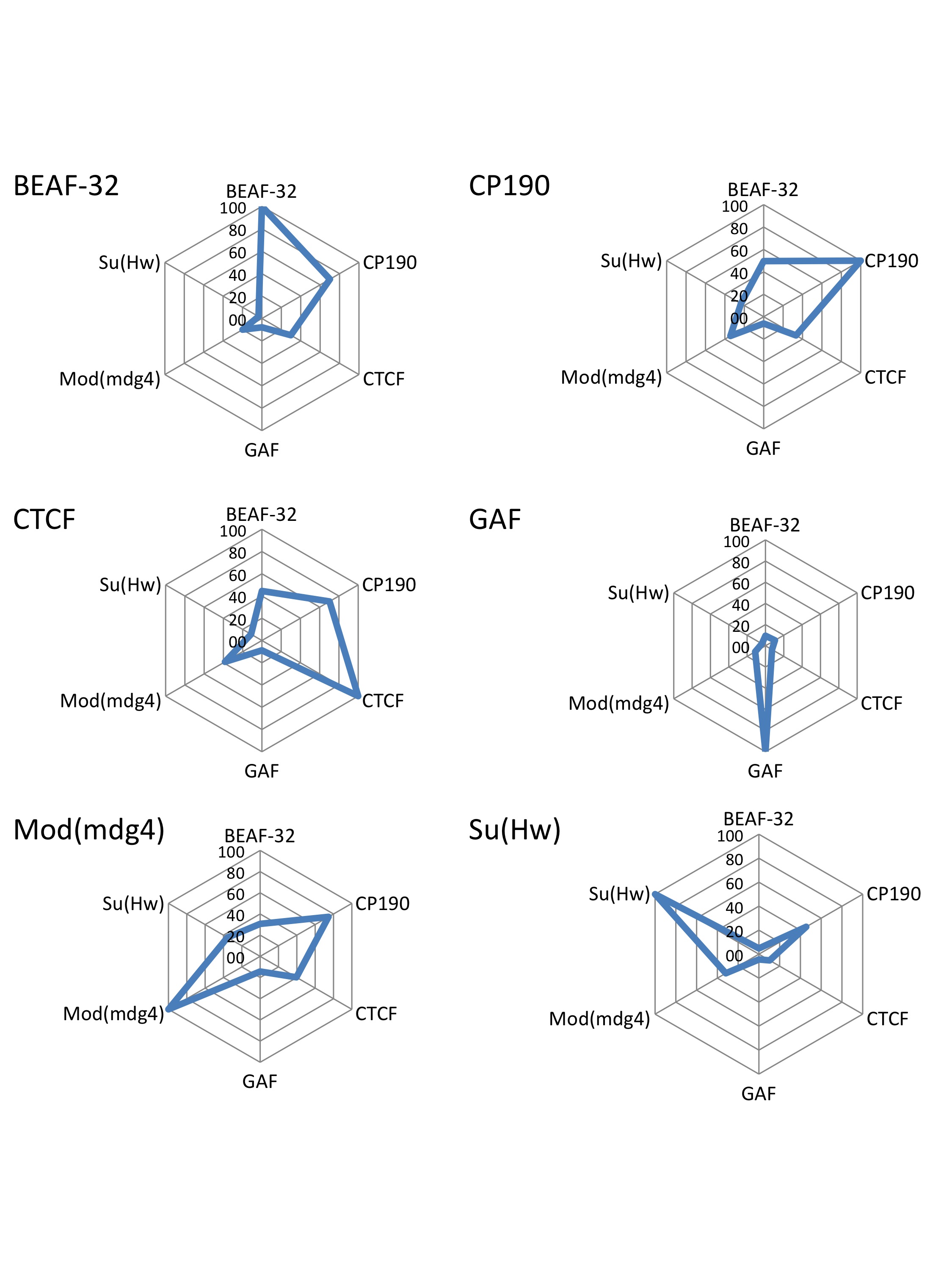

Supplement: Figure S3 — Overall pair-wise comparison between different factors. The axes in the radar plots indicate the percentage of overlapping binding site for one factor compared to each of the other factors. Data for CTCF and Su(Hw) corresponds to the CTCF_C and Su(Hw)-1 datasets respectively. This representation allows a quick identification of the preference of association between factors. For example, GAF is principally associated with itself and no other factor, while CTCF overlaps to a greater extent with CP190, Mod(mdg4), and BEAF-32, but not with GAF and Su(Hw). (0.53 MB JPG) [file pgen.1000814.s003.jpg]

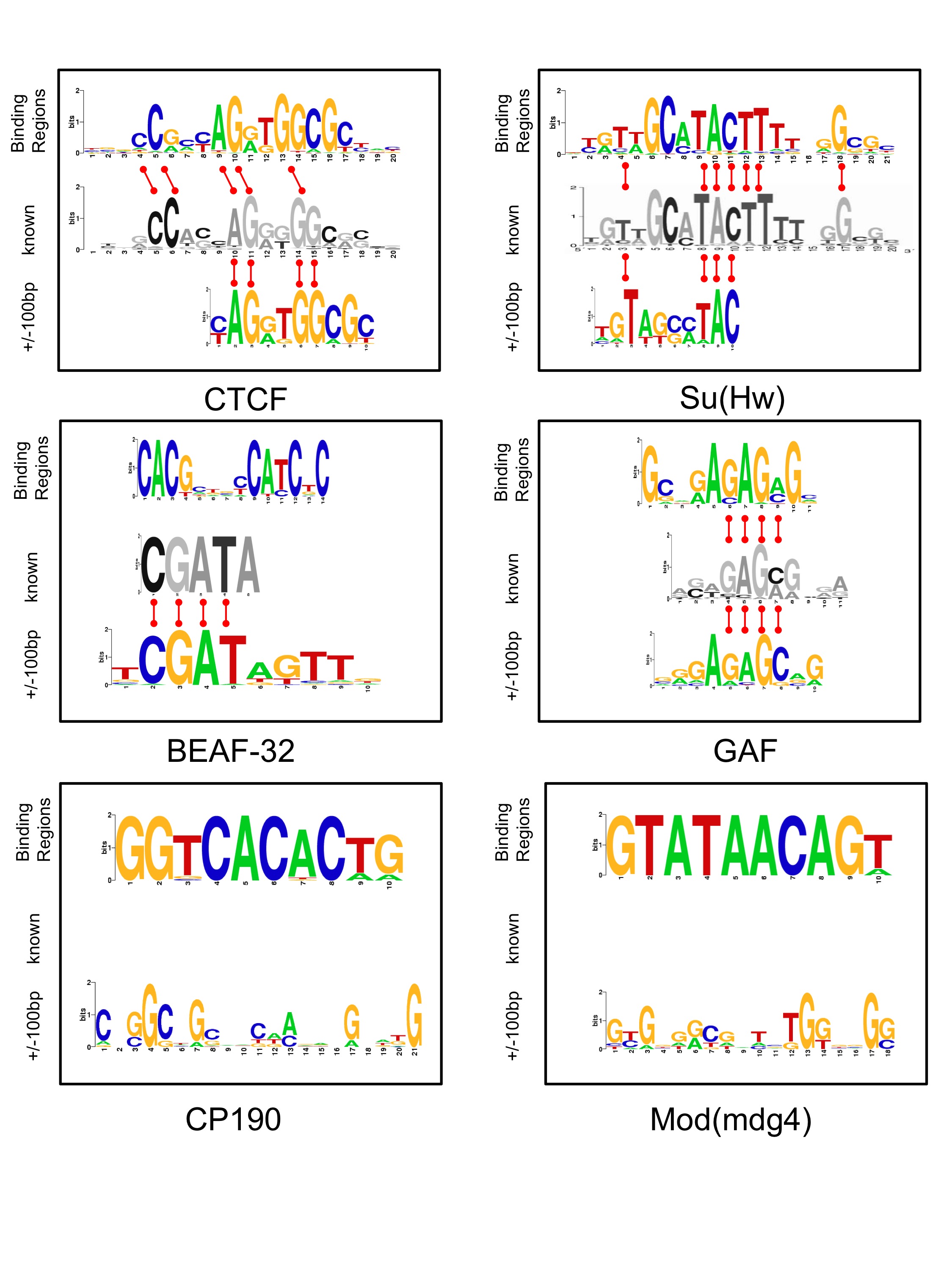

Supplement: Figure S4 — de novo Identification of DNA motifs. The newly discovered motifs for each factor are represented in color logos, while the previously known motifs are represented in gray scale. We present the motifs corresponding to 2 different discovery regions: the original peak regions as called by MAT (noted Binding Regions; median size ∼1,000 bp) and ±100 bp around the center of each peak (see Materials and Methods). The newly discovered motifs for CTCF, Su(Hw) and GAF are in agreement with previously described motifs [8],[10],[20], while the motif discovered for BEAF only agrees with previous studies [21],[22] when discovery is performed using the smaller ±100 bp regions. Interestingly, using the larger MAT regions, high information content motifs are identified for both CP190 and Mod(mdg4) which are not thought to bind DNA directly. The CP190 motif matches a known Vertebrate centromeric sequence [23]. However, the top motifs discovered using the ±100 bp regions are highly degenerate suggesting that while the factors may not bind the DNA directly, co-factors might bind in the more distant vicinity of their peaks. (0.75 MB JPG) [file pgen.1000814.s004.jpg]

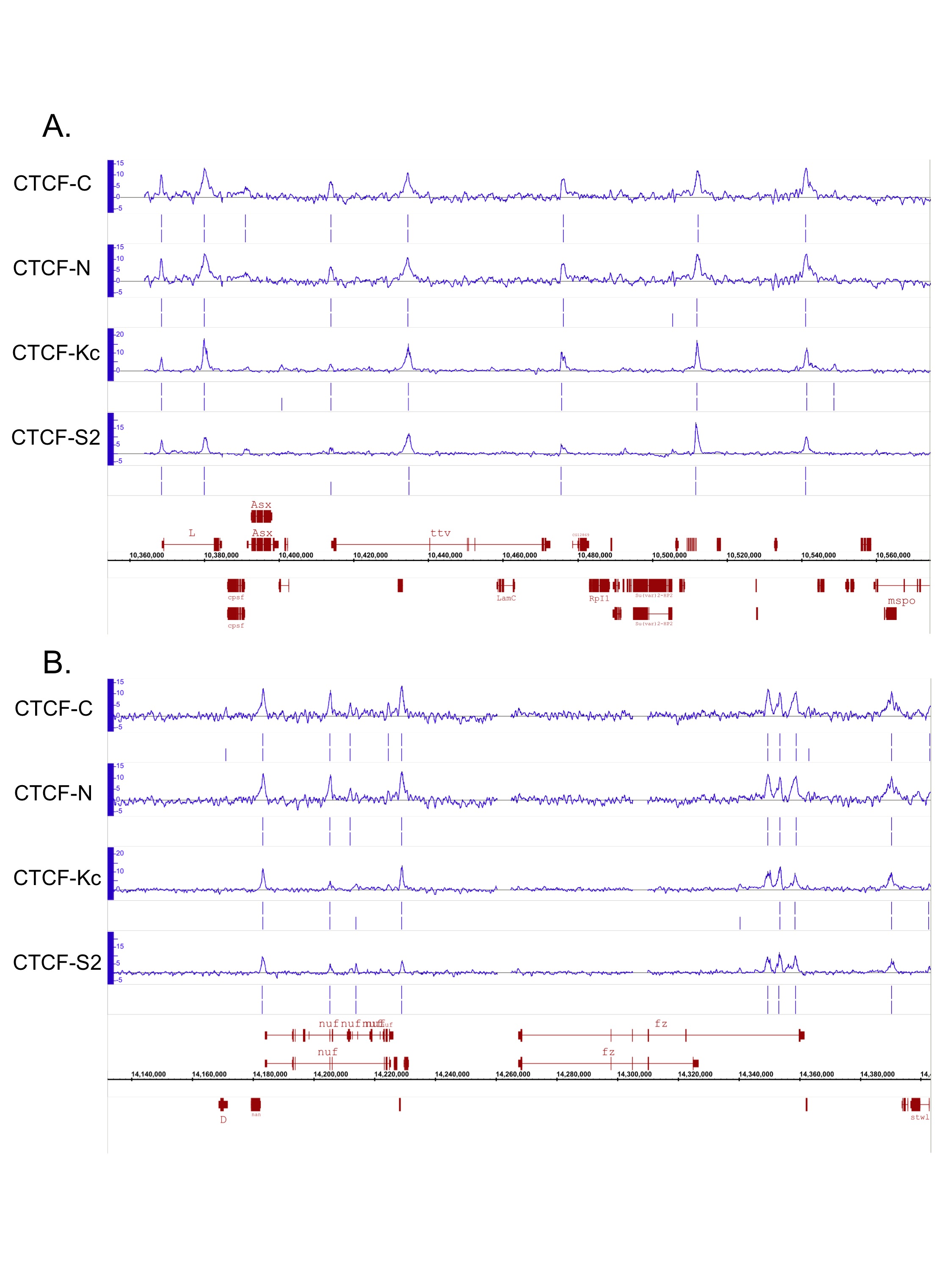

Supplement: Figure S5 — CTCF is a constitutive feature of the Drosophila genome. (A,B) In these genome browser views the ChIP-chip profiles for CTCF-C and CTCF-N in embryos are represented as top two tracks. Also represented are the ChIP-chip profiles for CTCF-N in two different cell lines: S2 cells and Kc cells. (0.62 MB JPG) [file pgen.1000814.s005.jpg]

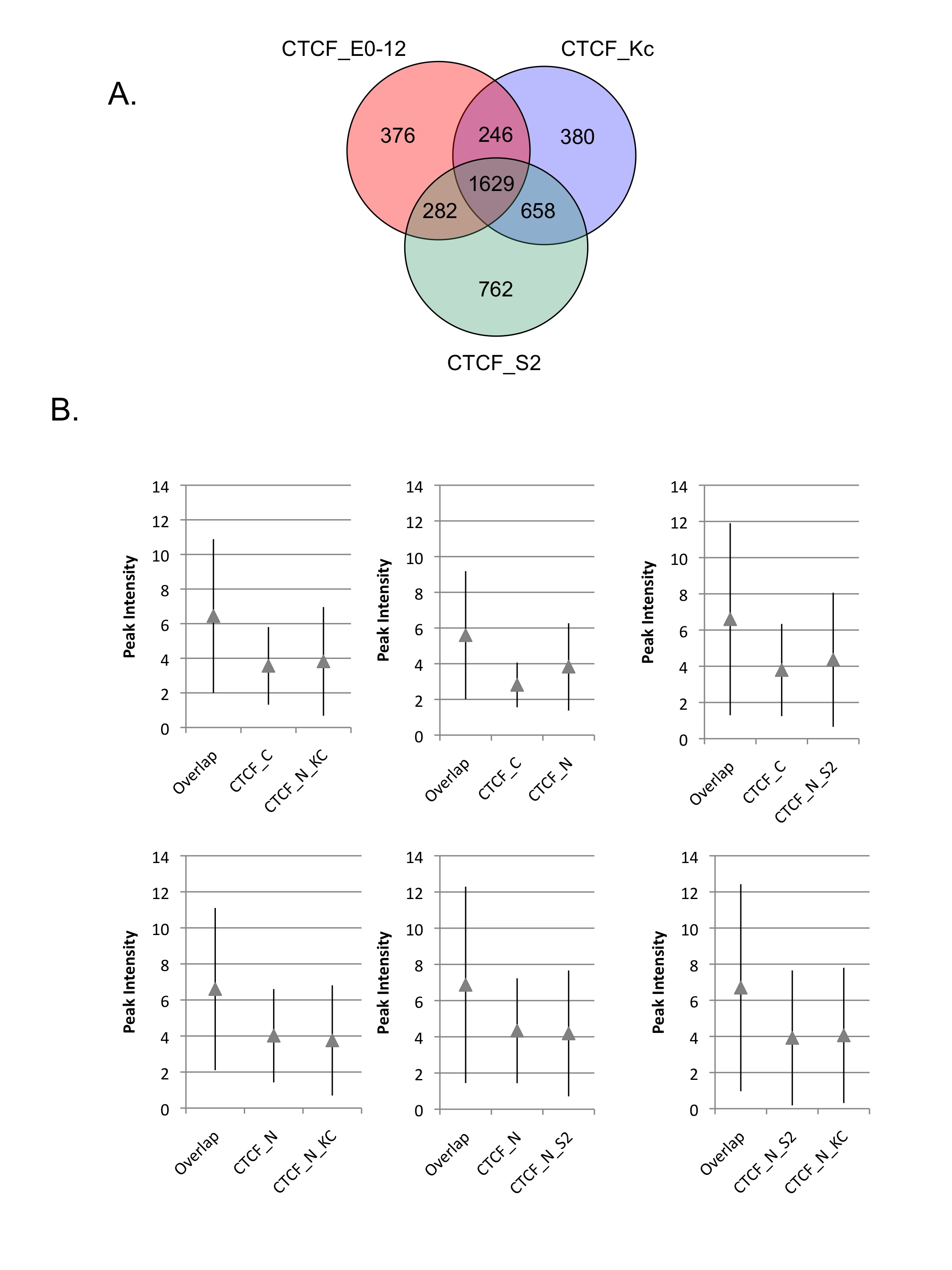

Supplement: Figure S6 — Decreased signal intensity at cell-type specific CTCF binding sites. (A) A Venn diagram showing the overlap between the binding sites for CTCF in embryos, in S2 cells and Kc cells. (B) The mean and standard deviation of the fold change for each pair-wise comparison between CTCF-C [embryos] and CTCF-N [embryos, S2 cells, Kc cells] is plotted for the peaks that do overlap, and the peaks that don't. The same statistical criteria applied to different datasets might not represent the variation between the different biological samples. (0.39 MB JPG) [file pgen.1000814.s006.jpg]

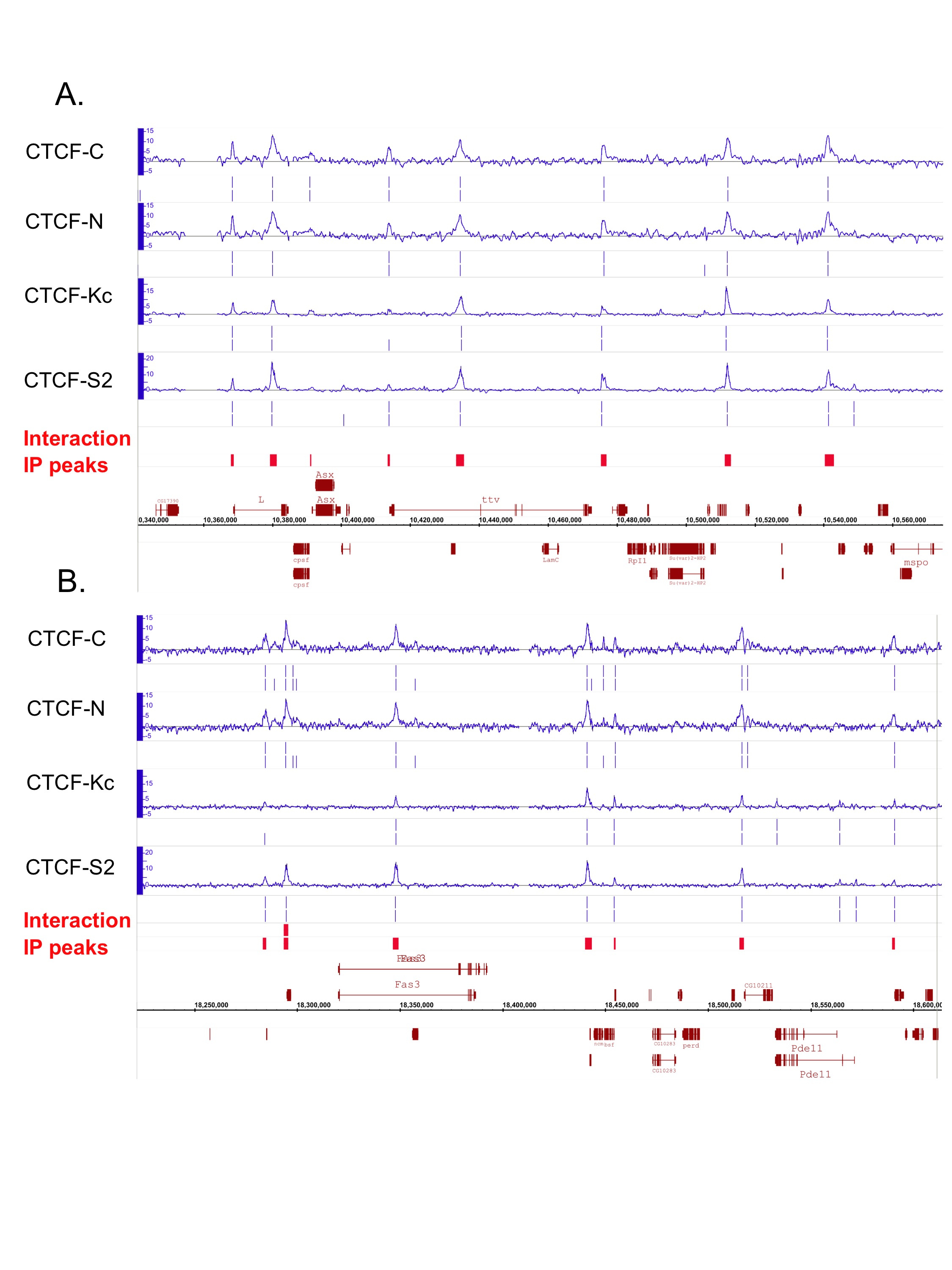

Supplement: Figure S7 — A joint-model analysis of the binding sites of CTCF in different tissues. All the raw data from CTCF ChIP-chip in different tissues have been analysed together with a joint model (see Text S1). A p value corresponding to 1% FDR has been applied to identify the binding sites. The same p value threshold has been applied to estimate the statistical difference of a peak in one condition compared to the others. (A,B) A comparative genome browser view of the results obtained by the joint model and a MAT analysis. In the first example (A) no difference is detected among the 3 profiles, while in (B) a binding site for CTCF upstream of the Fas3 gene is absent in Kc cells. (0.65 MB JPG) [file pgen.1000814.s007.jpg]

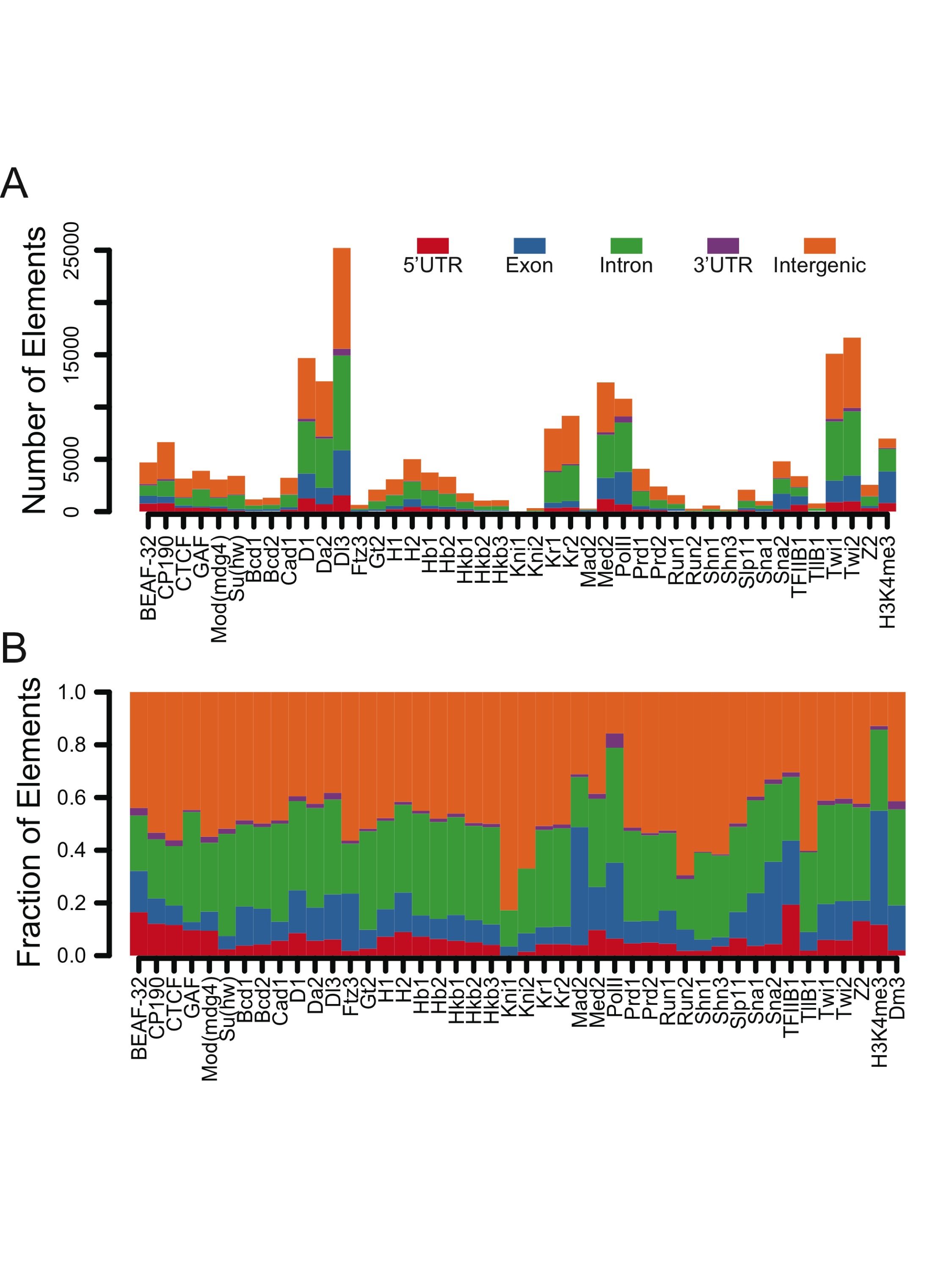

Supplement: Figure S8 — Distribution of the different classes of insulator binding sites compared to genomic features of Drosophila. (A) Barchart indicating the number of insulator binding sites of each class mapping to 5′ UTRs (red), exons (blue), introns (green), 3′ UTRs (purple), and intergenic regions (orange). For comparison, this distribution is also plotted for the set of transcription factors from MacArthur et al. [24] and for H3K4me3. (B) Data as in (A) normalized within each class to illustrate the fraction of insulators mapping to each annotation type. Also plotted at the right of the graph is the percentage of each region present in the Dm3 assembly of the Drosophila genome. (0.57 MB JPG) [file pgen.1000814.s008.jpg]

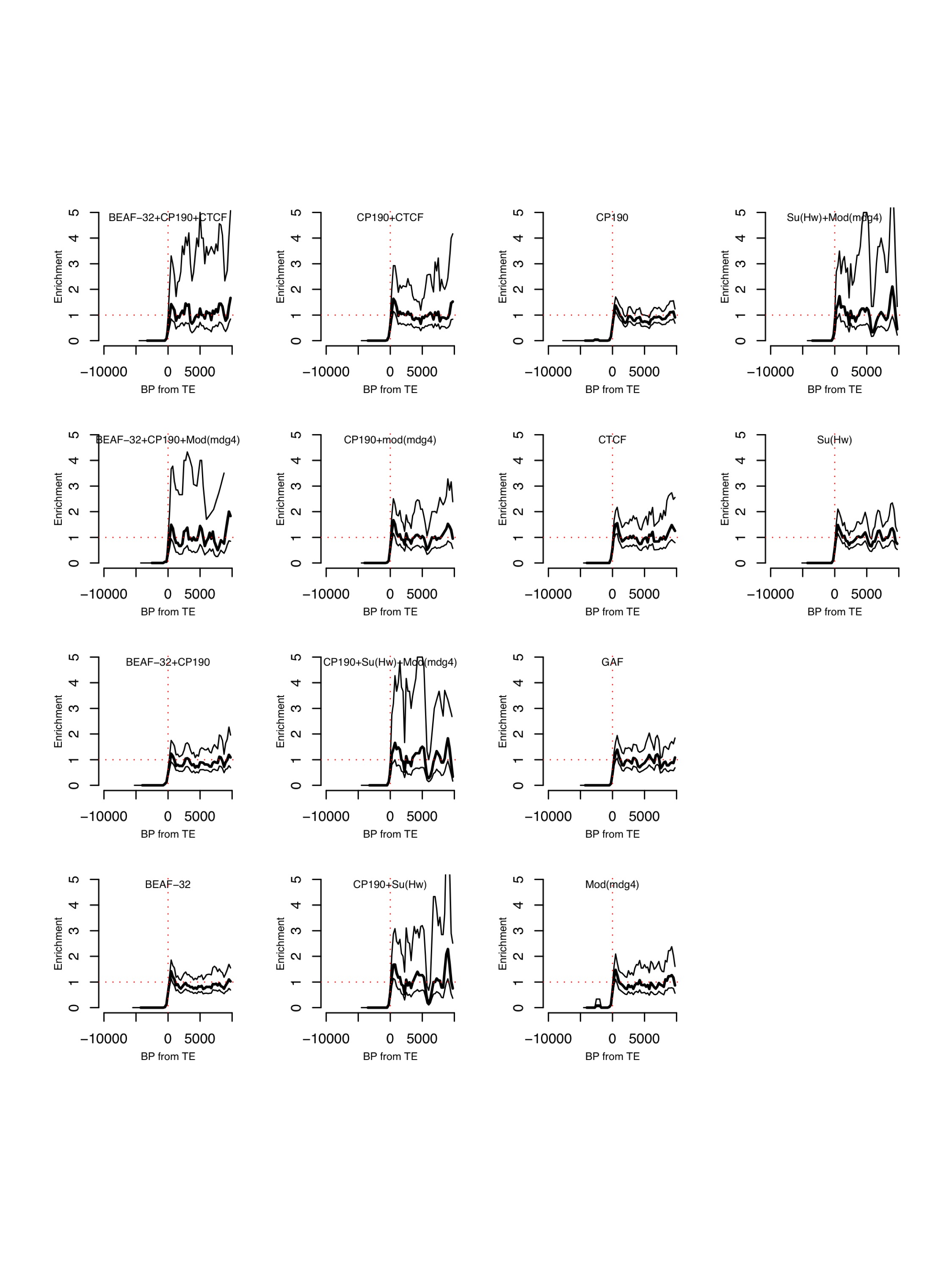

Supplement: Figure S9 — Distribution of the distance of insulator proteins binding sites relative to Transposable Elements. Estimated enrichment of insulator binding sites (black lines), with flanking 95% confidence intervals (gray lines) (Y-axis) are plotted against binding site base pair position (x-axis), relative to transposable element boundaries. Negative positions indicate binding sites within an annotated transposable element, 0 indicates the element boundary, and positive values represent positions outside and flanking element annotations. (0.53 MB JPG) [file pgen.1000814.s009.jpg]

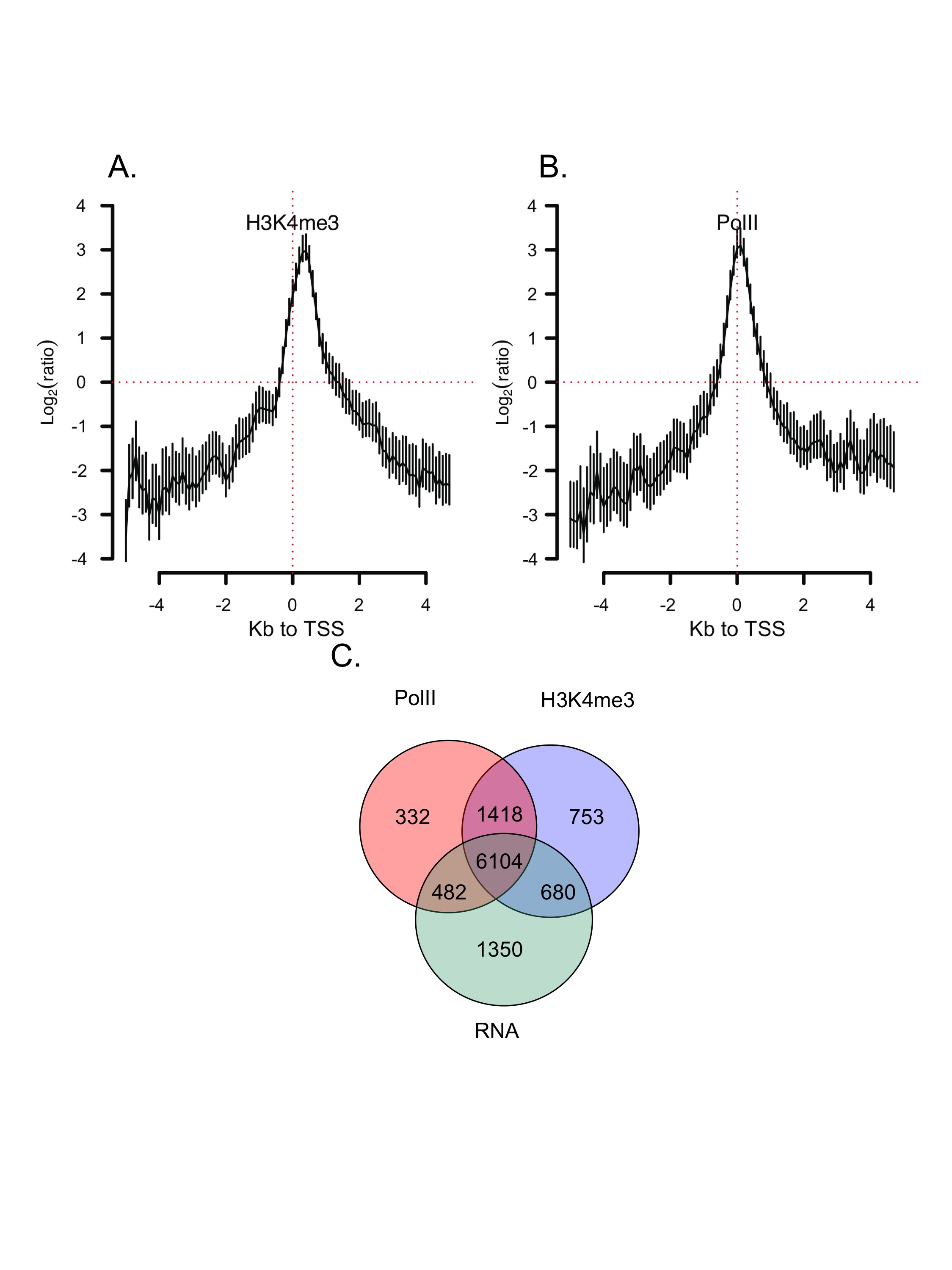

Supplement: Figure S10 — Expression status of Drosophila embryos. (A,B) Enrichment and 95% confidence intervals (Y-axis) plotted against distance to transcription start sites (x-axis) for identified PolII enriched regions (A) or H3K4Me3 enriched regions (B). (C) Venn Diagram representing genes associated with a PolII binding sites at their TSS, an H3K4me3 mark at their TSS and a RNA signal on their exon. (0.34 MB JPG) [file pgen.1000814.s010.jpg]

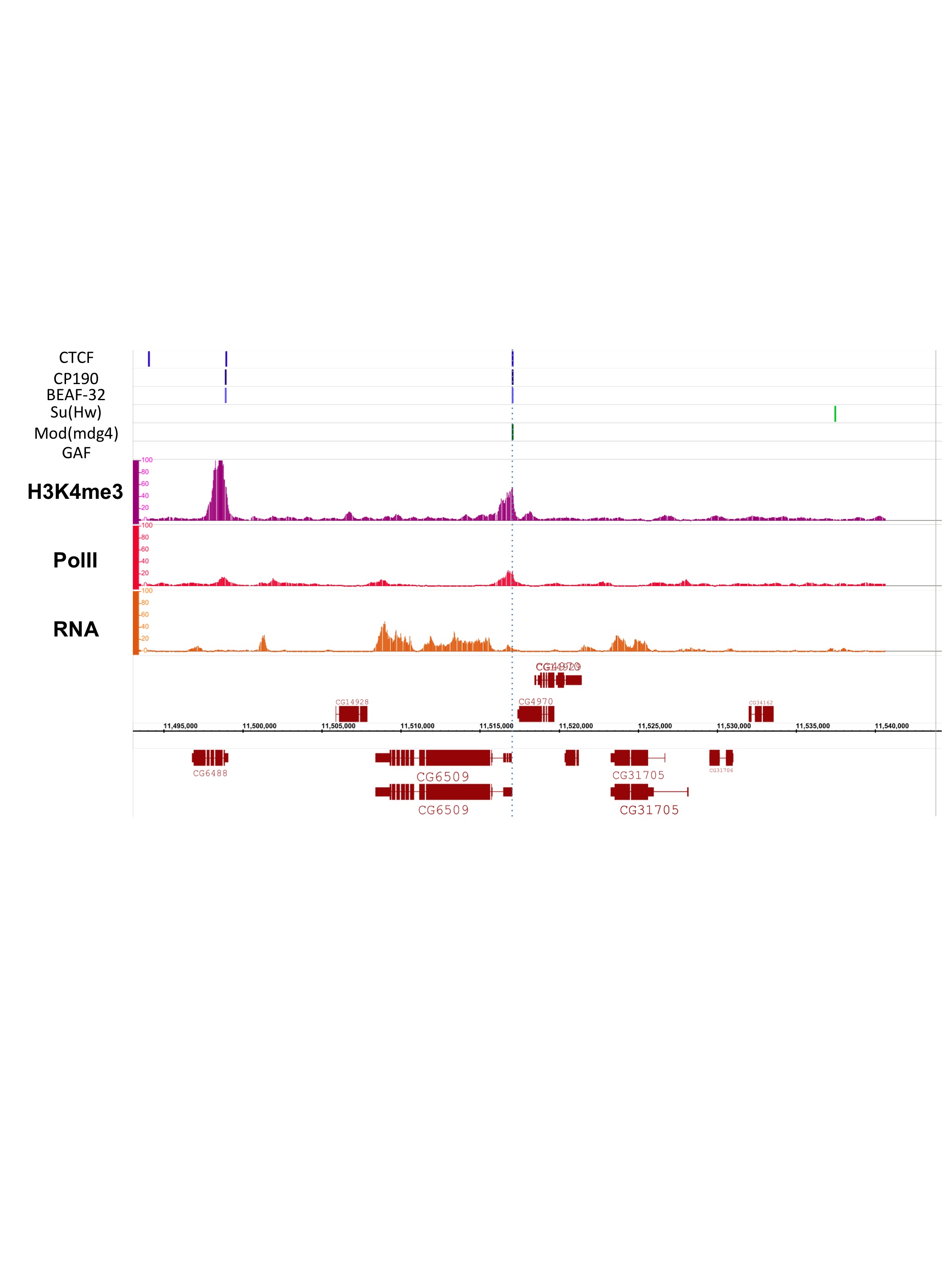

Supplement: Figure S11 — Example of position of insulator binding sites at divergent promoters. A genome browser example of signal obtained by ChIP-chip for H3K4me3 (purple), PolII (red), as well as total RNA profiling on tiling microarrays (orange). Insulator binding sites are also represented in this example where we can observe that a Class I insulator, defined by the binding of CTCF, CP190, BEAF-32, and Mod(mdg4), is located between the divergent genes CG6509 and CG4970 which are separated by approximately 350 bp. CG6509 is transcribed as identified by its RNA level and have an active promoter, as identified by the presence of PolII and H3K4me3 at its TSS. CG4970, however, is inactive, thus suggesting that the presence of the insulator allows CG4970 to be activated independently of CG6509. (0.31 MB JPG) [file pgen.1000814.s011.jpg]

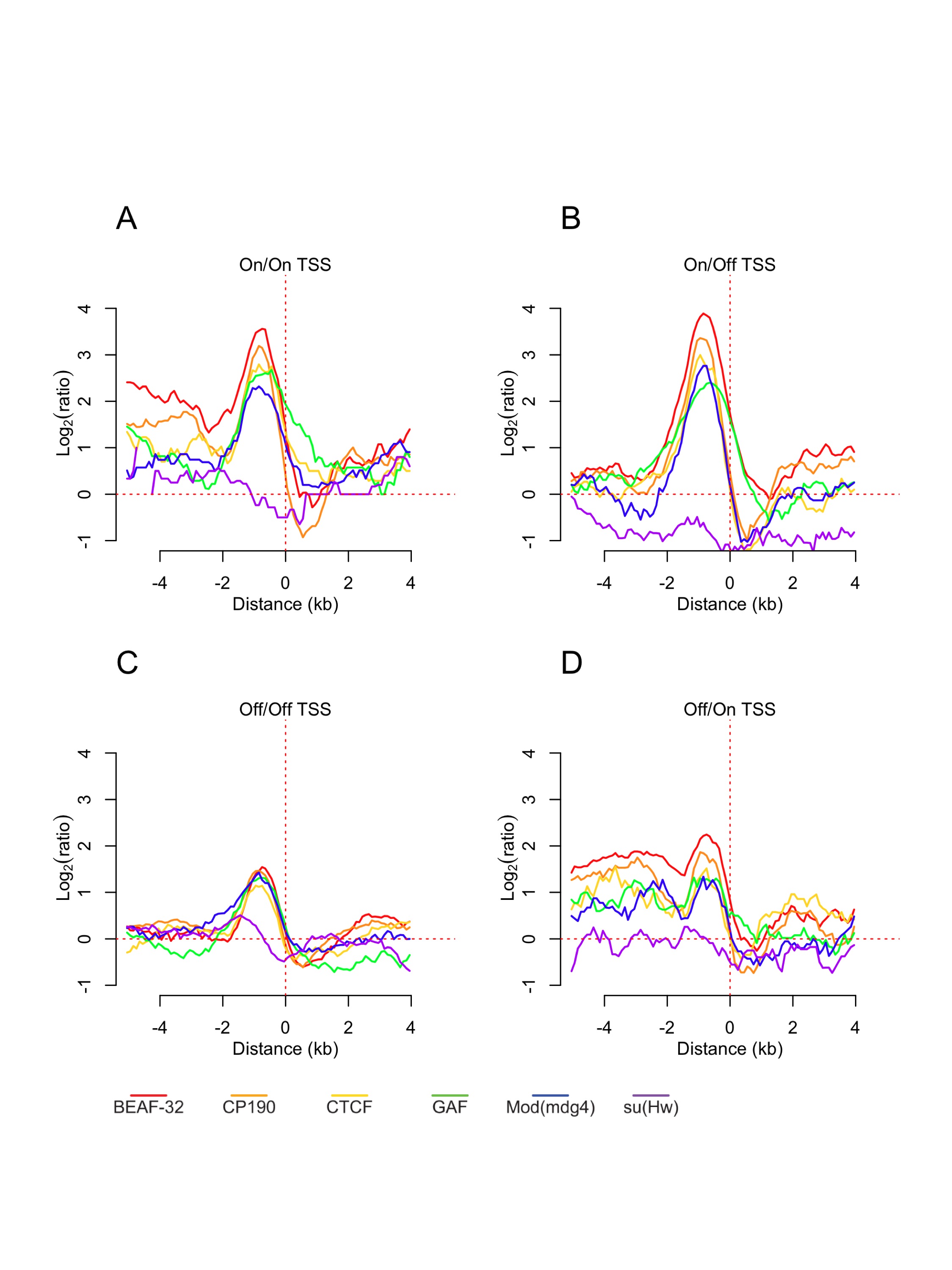

Supplement: Figure S12 — Distribution of insulator binding sites around the TSS of genes dependent of their transcription status. Log enrichment or depletion of insulator binding sites (y-axis) are plotted against binding site base pair position (x-axis), relative to the transcription start sites; negative and positive values depict upstream and downstream binding, respectively. Each panel corresponds to cases where the promoter is either active (On) or inactive (Off), as defined by the presence or absence of H3K4me3 and PolII (Figure S10C) and the transcriptional status of the nearest upstream promoter. (A) The gene TSS is on and the nearest upstream TSS is on. (B) The gene TSS is On and the nearest upstream TSS is Off. (C) The gene TSS is Off and the nearest upstream TSS is Off. (D) The gene TSS is Off and the nearest upstream promoter is On. (0.50 MB JPG) [file pgen.1000814.s012.jpg]

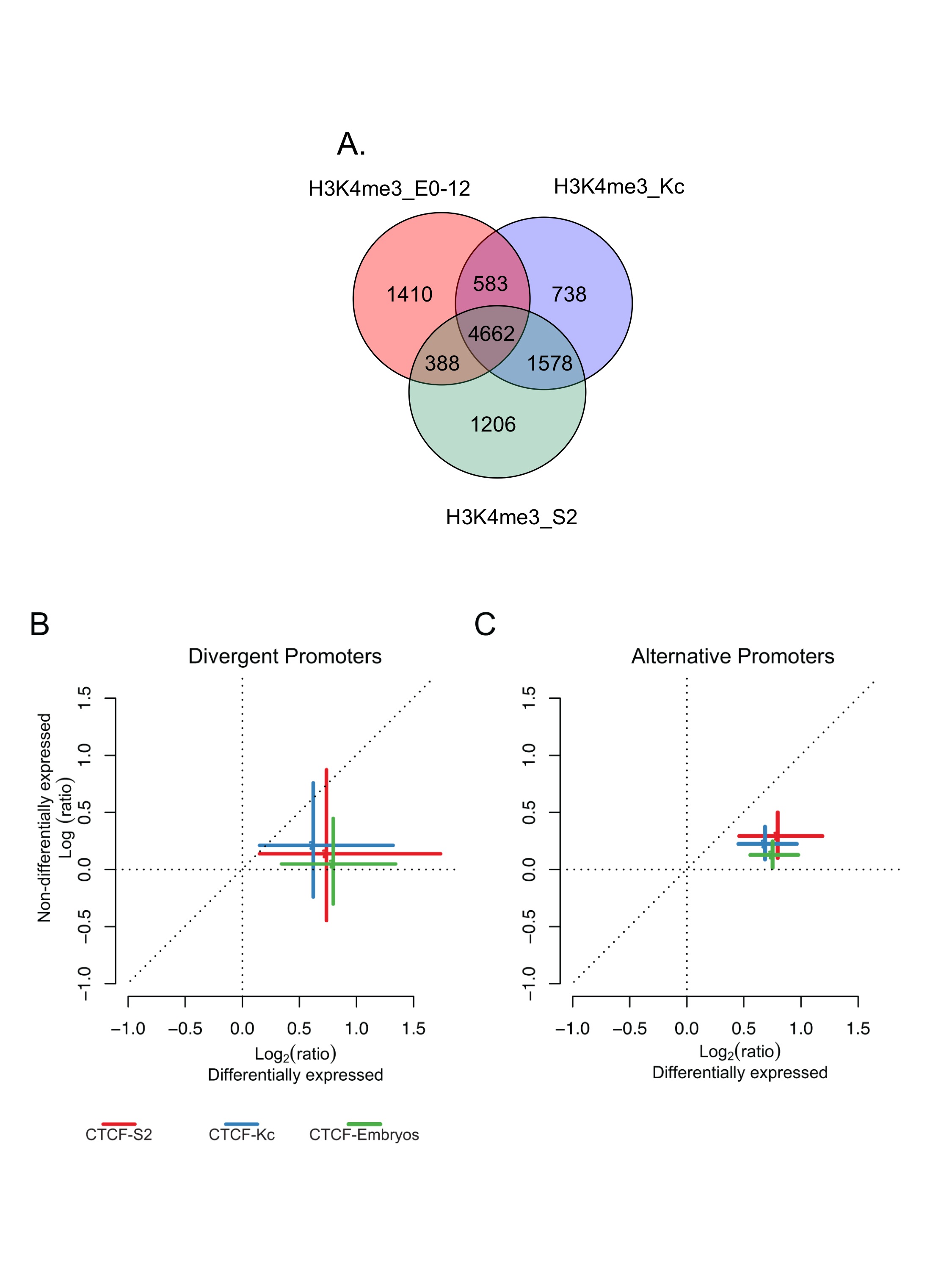

Supplement: Figure S13 — Enrichment of CTCF binding sites between Adjacent Promoters in different cell lines. (A) Venn diagram between H3K4me3 associated promoters in embryos, S2 cells and Kc cells. Enrichment and 95% confidence intervals of CTCF binding sites in embryos, S2, and Kc cells between (B) divergent and (C) alternative promoters. (0.34 MB JPG) [file pgen.1000814.s013.jpg]

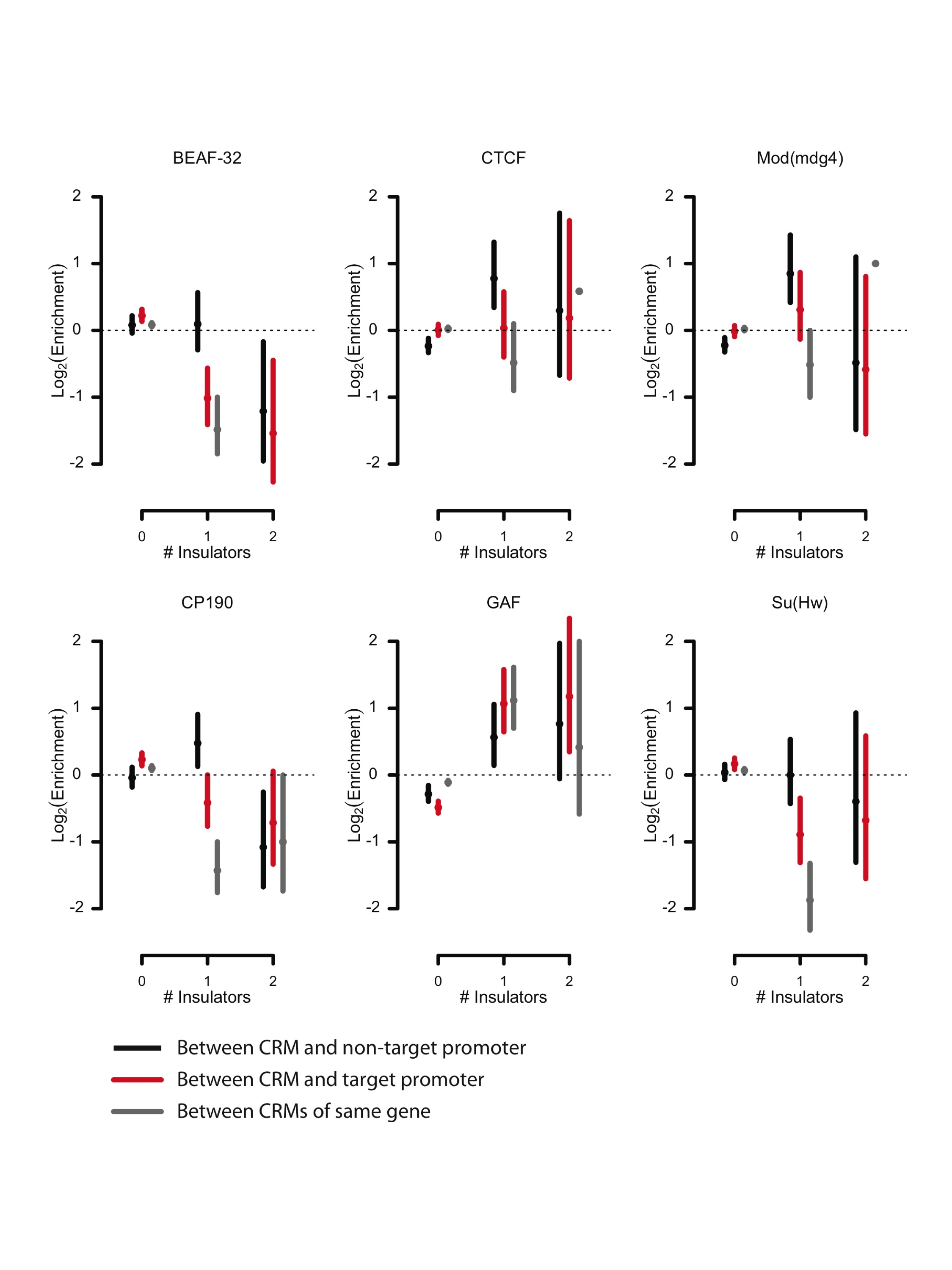

Supplement: Figure S14 — Enrichment of different classes of binding sites between CRMs and Promoters. For each insulator binding site class, enrichment estimates and flanking confidence intervals (Y-axis) are plotted for genomic intervals with 0, 1, or 2 insulator binding sites (X-axis). Intervals are defined by the region between cis-regulatory elements and their target promoters (red), or between cis-regulatory elements and their nearest non-target promoters (black), or between adjacent cis-regulatory elements that regulate the same gene (gray). (0.36 MB JPG) [file pgen.1000814.s014.jpg]

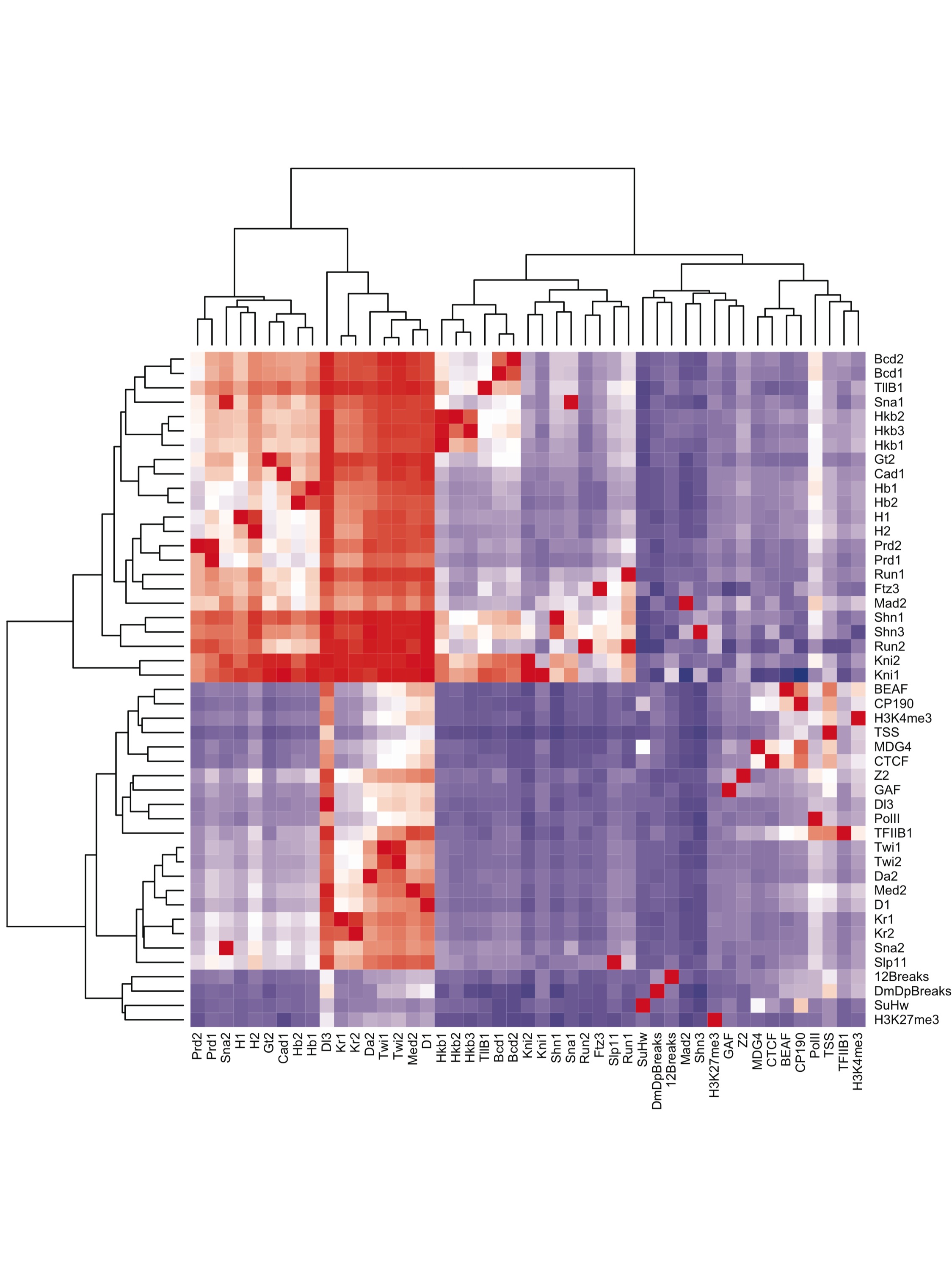

Supplement: Figure S15 — Insulator proteins do not associate with transcription factors binding sites. Binding sites from 36 datasets corresponding to 21 transcription factors [24] were downloaded from UCSC and compared to our set of insulator binding sites. A distance matrix was constructed as 1 minus the fraction of sites with midpoint to midpoint distances less than 250 bases (data in Table S4) and hierarchically clustered using the average linkage method. Cell colors range from blue to white to red to depict increasing site overlap. (0.59 MB JPG) [file pgen.1000814.s015.jpg]

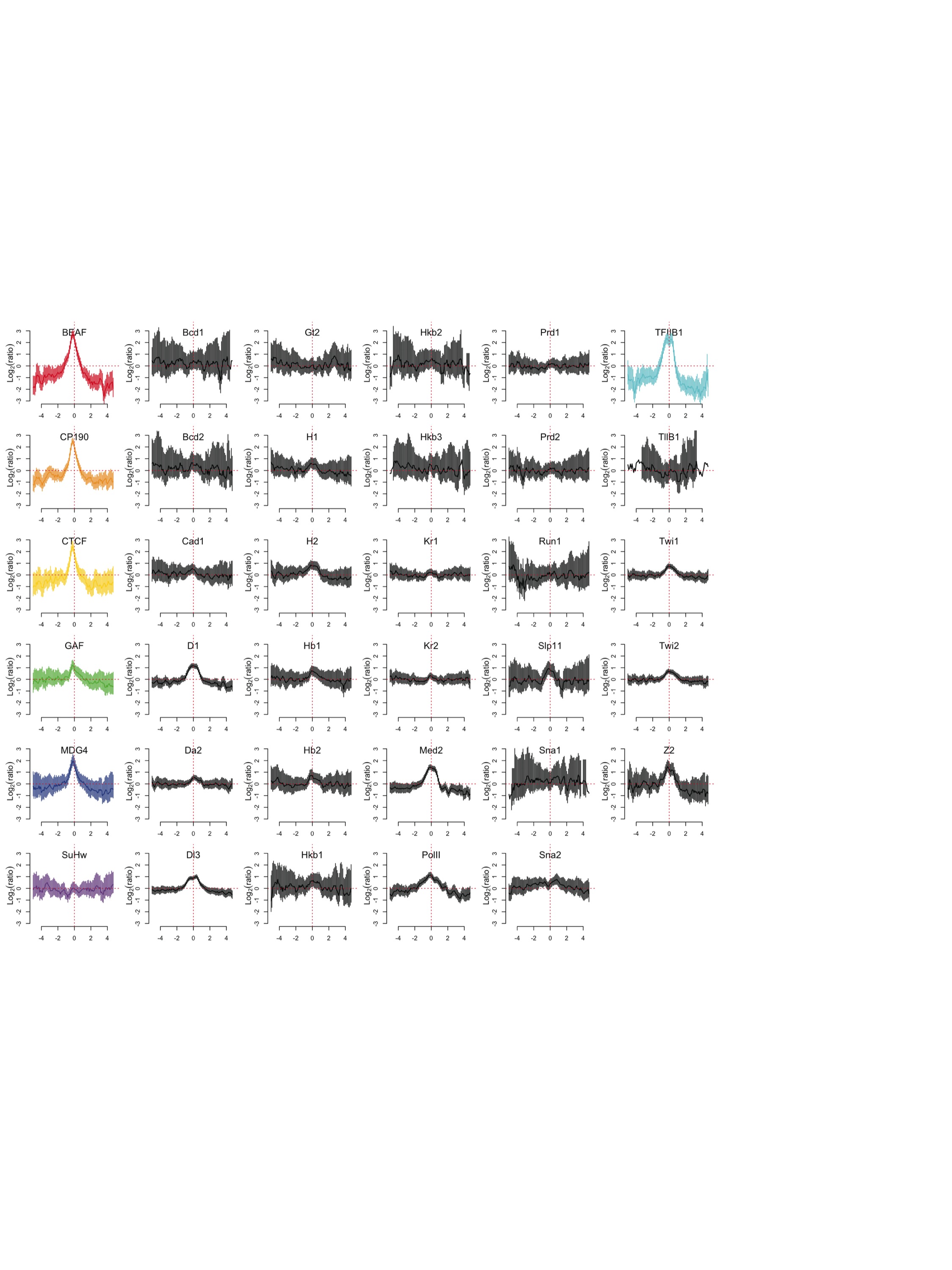

Supplement: Figure S16 — Enrichment at the TSS of insulator proteins and transcription factors. Binding sites from 36 datasets corresponding to 21 transcription factors (BDTNP; [24]) were downloaded from UCSC and compared to our set of insulator binding sites. Log2 enrichment or depletion of insulator binding sites and associated 95% confidence intervals (Y-axis) are plotted against binding site base pair position (X-axis), relative to the transcription start sites; negative and positive values depict upstream and downstream binding, respectively. A point of comparison for a promoter associated factor TFIIB is represented in light blue. (0.59 MB JPG) [file pgen.1000814.s016.jpg]

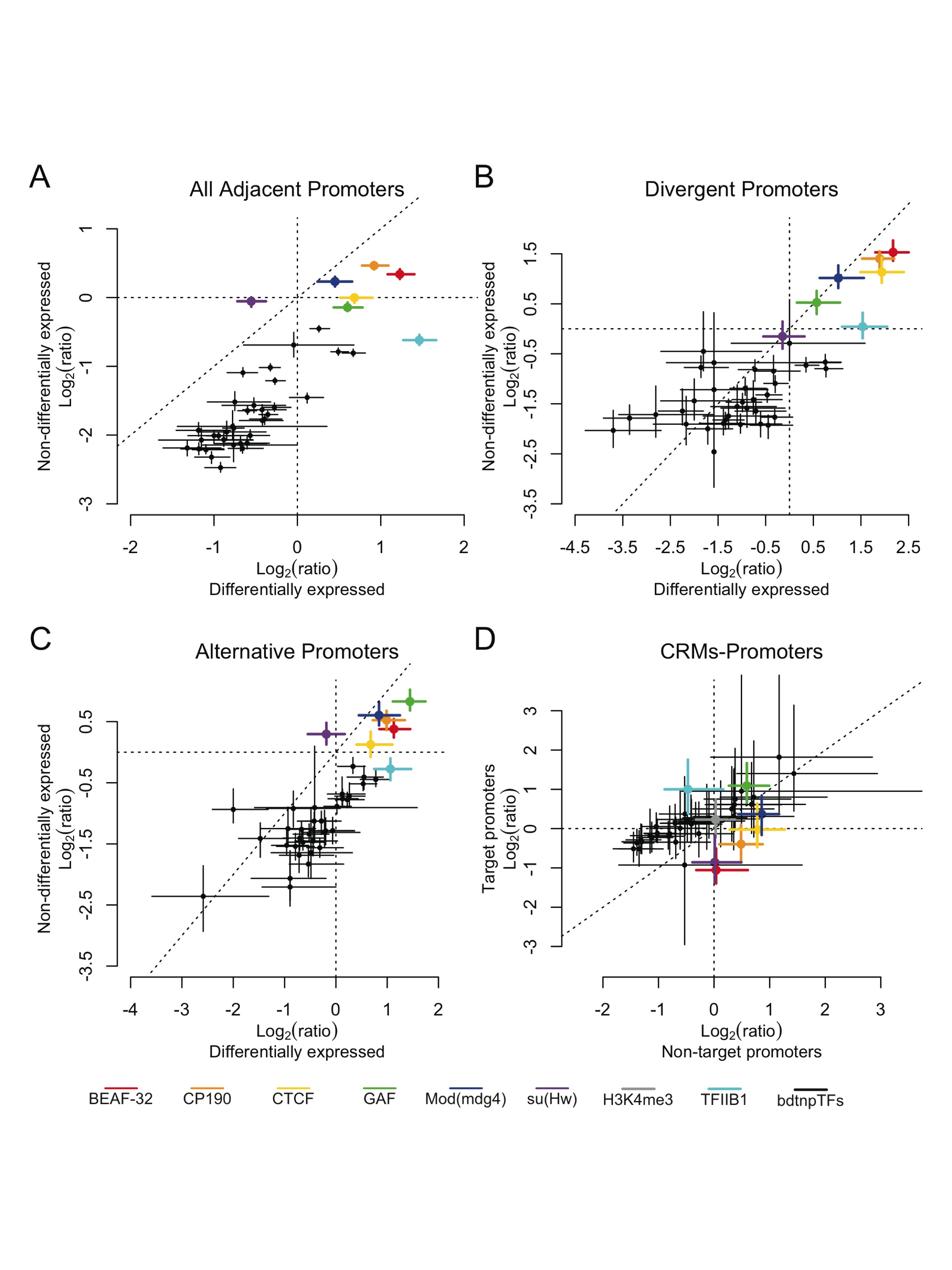

Supplement: Figure S17 — Promoter demarcation by insulators and transcription factors. Same Legend as in Figure 4. Now represented in black are the data corresponding to the BDTNP datasets. TFIIB is represented in light blue. (0.53 MB JPG) [file pgen.1000814.s017.jpg]

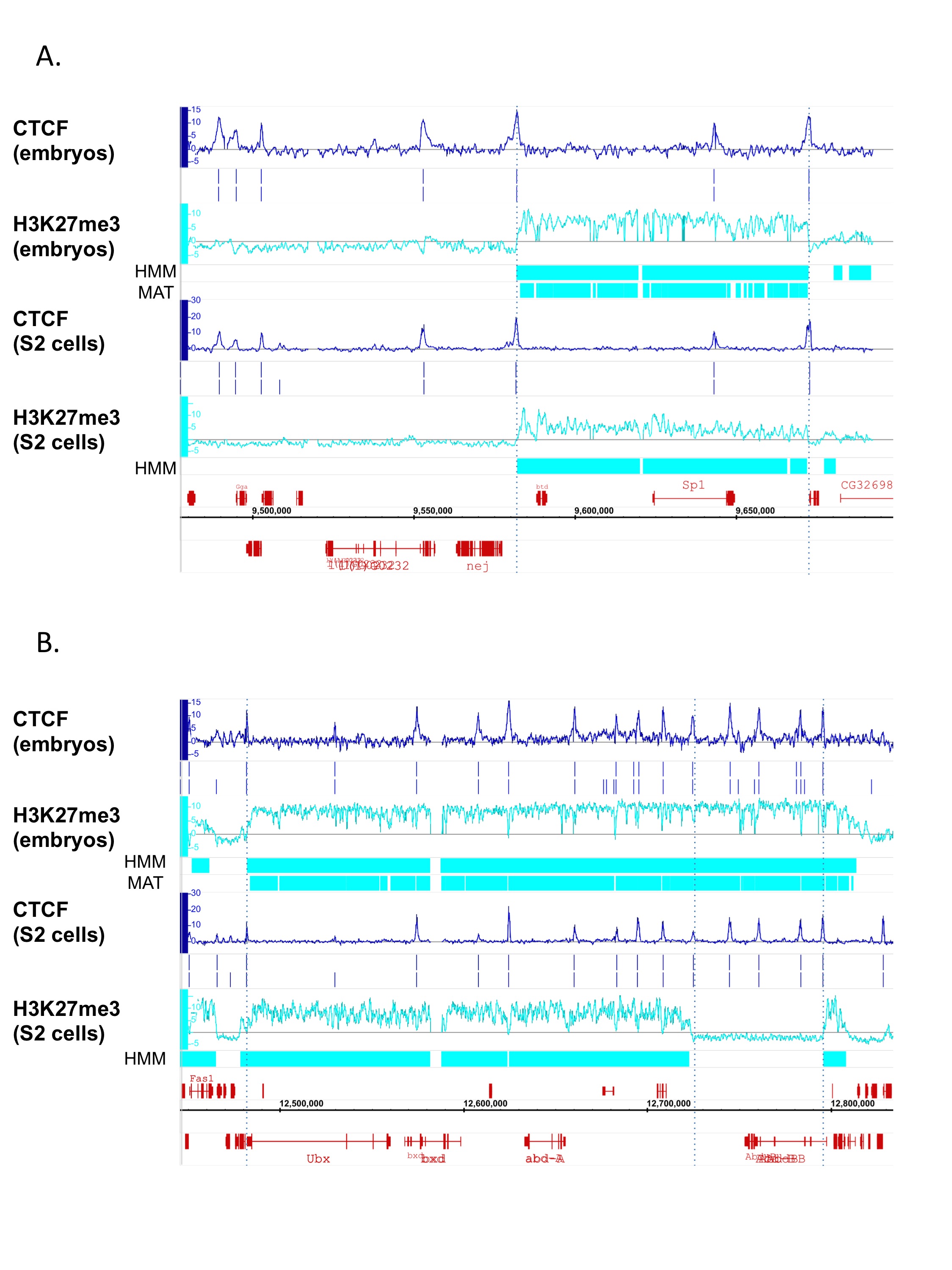

Supplement: Figure S18 — CTCF at the boundaries of H3K27me3 domains. Genome browser example showing signals for CTCF and H3K27me3 ChIP-chip experiments performed in embryos and S2 cells. The H3K27me3 data in S2 cells is reanalyzed from [3] (see Text S1). HMM segmentation is used to define the boundaries of H3K27me3 better. This can be visualized on these examples when compared with a MAT analysis performed on embryos. The dashed vertical lines show CTCF binding sites at domain boundaries. (A) Example of CTCF bordering an H3K27me3 domain covering the btd/Sp1 locus. (B) In this example, an entire H3K27me3 domain corresponding to the Abd-B gene disappears, while this chromatin mark is maintained in the rest of the Bithorax Complex region. This depletion of the H3K27me3 mark corresponds to Abd-B being expressed in S2 cells while Ubx and abd-A are repressed. Embryos corresponding to a mixed population of cells, the H3K27me3 signal is coming from its presence in a subpopulation of cells. All CTCF binding sites in this region are conserved between S2 cells and embryos, even inside the H3K27me3 depleted domain (between the dashed vertical lines) and are therefore independent of the transcriptional status of Abd-B. Furthermore, the breakpoints of the H3K27me3 depleted domain in S2 cells, compared to embryos, correspond to CTCF binding sites (represented by the two vertical dashed lines). (0.78 MB JPG) [file pgen.1000814.s018.jpg]

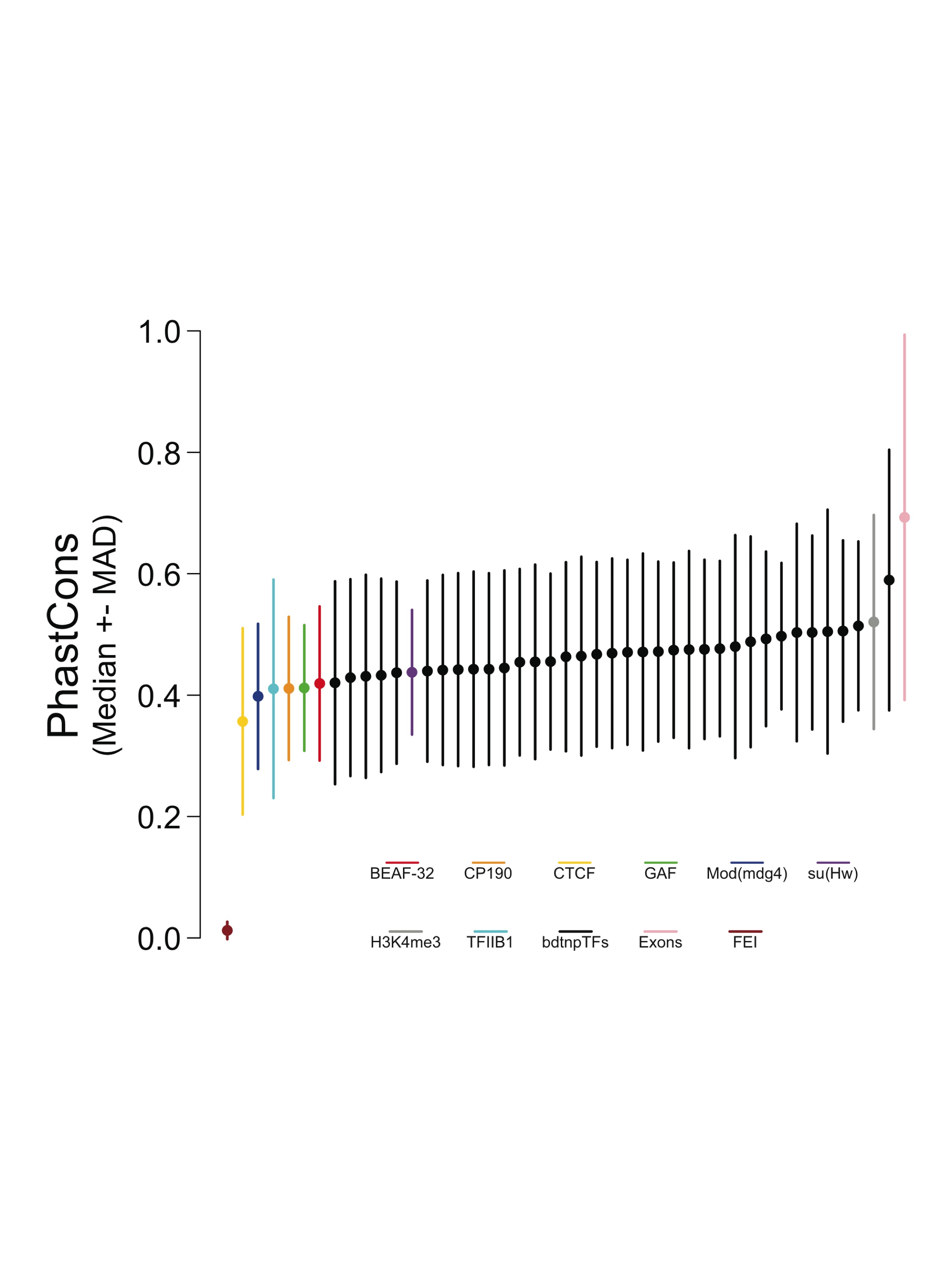

Supplement: Figure S19 — Conservation of insulator binding sites. Phastcons between 15 insect species, including the 12 sequenced Drosophilae species, have been calculated for each category of insulator binding sites. The bars correspond to the median (dot) and median absolute deviation (bars) of the scores. The dark red bar (FEI) corresponds to the same scores calculated for fast evolving introns (neutral reference). Also plotted for reference are exons (pink), H3K4me3 (gray), and BDTNP binding sites (black). (0.30 MB JPG) [file pgen.1000814.s019.jpg]

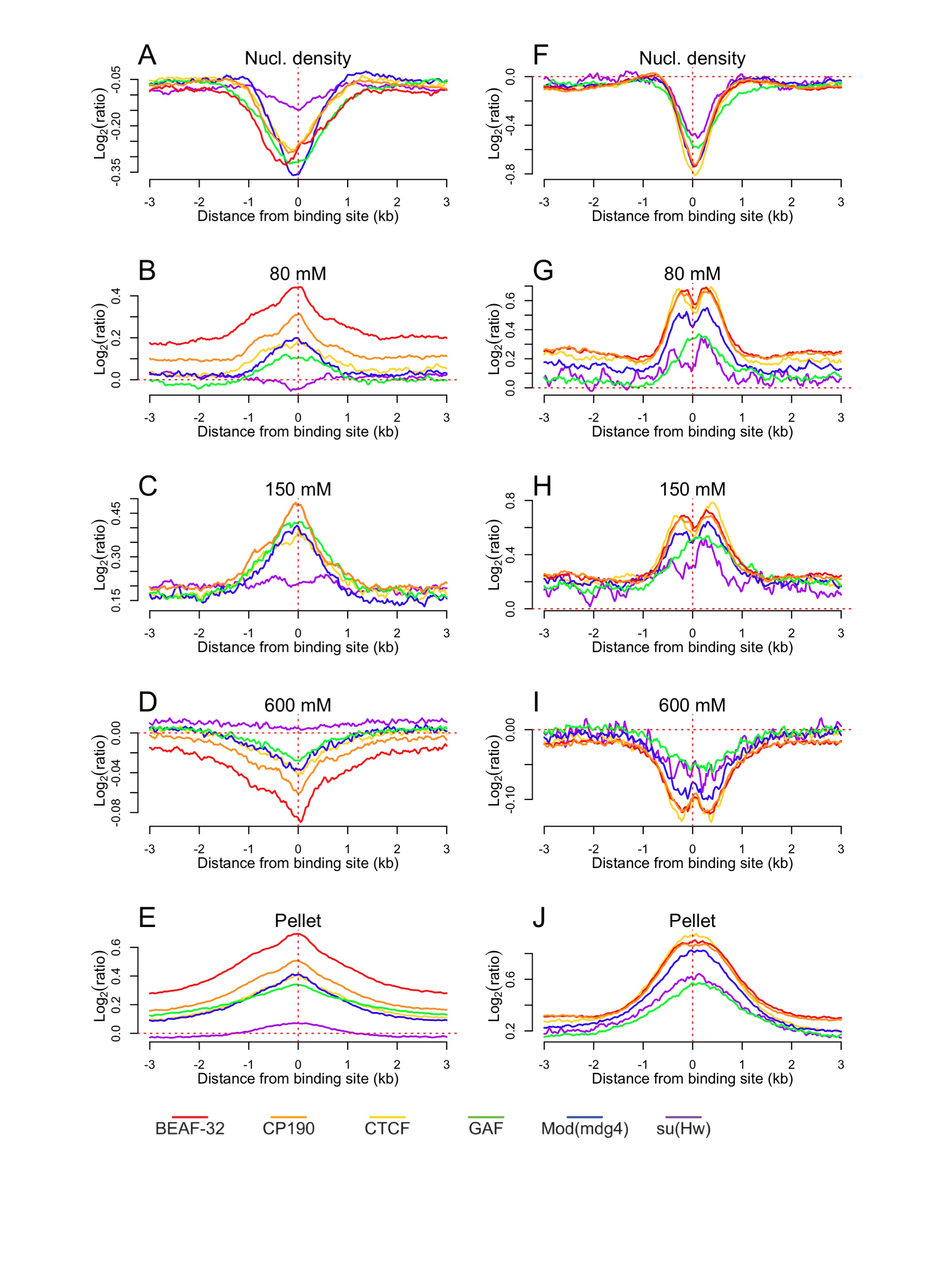

Supplement: Figure S20 — Dynamic chromatin at insulator binding sites at non-promoter and promoter sites. Each insulator site, defined as the midpoint of the binding site interval, was classified as to whether it fell within the interval defined by a transcriptional start site and 500-bp upstream, using the 12,807 unique 5′ ends annotated in FlyBase r5.13. Based on these criteria, the number of sites in non-promoters and promoters are: BEAF-32 (5546 nonpromoters, 2281 promoters); CP190 (7758 non-promoters, 2698 promoters); CTCF (3286 non-promoters, 1146 promoters); Mod(mdg4) (3154 non-promoters, 821 promoters); GAF (5551 non-promoters, 887 promoters); Su(Hw) 4565 non-promoters, 214 promoters). Displays are for non-promoters (A-E) and promoters (F-J) using the same datasets shown in Figure 5. (A,F) Nucleosome density; (B,G) 80 mM salt fraction; (C,H) 150 mM salt fraction; (D,I) 600 mM salt fraction; (E,J) salt-washed pellet. (0.82 MB JPG) [file pgen.1000814.s020.jpg]
