# Supplementary material for: A Comprehensive Map of Insulator Elements for the Drosophila Genome
Source: PLoS Genet. 2010 Jan 15;6(1):e1000814. doi: 10.1371/journal.pgen.1000814 (PMC2797089; doi:10.1371/journal.pgen.1000814)
Supplement: Text S1 — Supplementary methods. (0.07 MB DOC) [file pgen.1000814.s025.doc]

**A comprehensive map of insulator elements for the *Drosophila* genome**

**Text S1. Supplementary Methods**

**Primary analysis of data**

**Analysis of ChIP-chip data on Affymetrix arrays**

We used Model based Analysis Tiling-arrays (MAT) software for the peak finding of the ChIP-chip data [1]. Array library files were mapped to current version (UCSC dm3) of the *Drosophila* genome assembly using xMAN [2]. We downloaded Repeat-masker and simple repeats information from UCSC genome browser http://hgdownload.cse.ucsc.edu/goldenPath/dm3/bigZips/ to generate a *Drosophila melanogaster* genome specific Repeat Library file to use with MAT. We ran paired MAT analysis with Bandwidth of 250, MaxGap value of 500, and MinProbe value of 10.

Genome-wide binding data for H3K27me3 in S2 cells [3] were downloaded from the ArrayExpress database [4].

**H3K27me3 domain identification**

We identified H3K27me3 domains as follows: .CEL files intensities were quantile normalized and mean values for replicate probes were calculated. Fold change for every probe on the array was then calculated and intensity information at every 100 base pair was identified with window based smoothing. This continuous intensity information was used as input to the Hidden Markov Model based segmentation for parameter learning and region calling. We used expectation maximization for parameter learning. Segmentation performance of the method was checked with multiple types of data normalization and window parameters. Most experiments resulted in identical segmentation illustrating robustness of the method. Details of the developed method can be found in [5-7].

**Analysis of Su(Hw) data (Adryan et al., Genome Biol, 2007)**

Nucleotide sequences corresponding to total 3402 Su(Hw) regions listed in Additional data file 1 in [8] were obtained using ENTREZ retrieval tool at the NCBI website (http://www.ncbi.nlm.nih.gov/sites/batchentrez?db=Nucleotide). These were remapped to the dm3 assembly using BLAT [9] and regions exhibiting a mean enrichment above 1.7-fold were used as Su(Hw) bound regions for overlap analysis.

**Analysis of CTCF data (Holohan et al., PLoS Genet., 2007)**

Co-ordinates corresponding to CTCF sites identified in [10] were remapped to dm3 assembly using *LiftOver* tool at the UCSC genome browser website (http://genome.ucsc.edu/cgi-bin/hgLiftOver) and used for overlap analysis.

**Clustering of insulator sites**

We identified different overlapping classes of insulators for all 6 insulators using at 250 bp as a distances threshold. We then hierarchically bi-clustered insulator class instances with different scores (identity, MAT score, fold change and negative log_10_ Pvalue for the peaks as identified by MAT) as feature vectors with different distance measures (Pearson’s absolute correlation, Spearman’s rank correlation and Kendell’s Tau) and different clustering paradigms (Pairwise complete-linkage, pairwise single linkage, pairwise centroid linkage as well as pairwise average linkage clustering) [11]. As expected, pairwise complete linkage clustering with Spearman’s rank correlation outperformed other methods with all distance measures in generating well formed clusters [12]. All other methods and distance matrices, apart from clustering runs with single-linkage clustering, in most cases gave topologies grouping (BEAF-32, (CP190, CTCF)), and (Mod(mdg4), Su(Hw)) together with GAF not falling in group with any other factors.

We also created an additional dataset by removing all single instances of insulators from the clustering input files to check for any bias that may be present due to large number of single peaks present in the input set. We obtained similar tree topologies.

**Identification of overlap between factors**

Overlap between factors were identified by calculating distance between 1) peak locations as suggested by MAT with a distance threshold; 2) using mid-point distance when peak location information wasn’t available and 3) physical overlap between enriched regions when working with large regions. We calculated overlaps at distance thresholds of 50 to 1000 with 50 bp increments and reported overlaps at distance threshold of 250 in our analysis.

For calculating overlap between 3 or more factors we implemented an algorithm that reads the combined peak (or mid point) information for those factors from 5’-to 3’ for each chromosome on the genome and searches for the next peak location falling within the distance threshold.

**Calculation of overlap significance for insulator overlap classes**

We ran 1000 simulations randomly assigning insulator peak positions for all 6 insulators on each chromosome of the genome as per their real distribution in the data. Total instances of overlapping insulator classes were calculated in each run at the distance threshold of 250 bp. A two-tailed P-value was calculated for each insulator class using its simulated distribution as the reference distribution.

**Depletion of insulators within the H3K27me3 domains**

Depletion of insulator occurrences within H3K27me3 domains was also examined with the simulation procedure similar to above. CP190 (2.1e-115) and BEAF-32 (2.4e-112), followed by CTCF, Su(Hw) and Mod(mdg4) - (~ 1e-9 to 1e-19) show significant depletion within H3K27me3 domains but GAF distribution is similar to the random distribution.

**Fold change differences in CTCF overlapping vs. non-overlapping sites.**

In order to check whether technical differences in experiments and subjective threshold of the binding site calls result in the loss of overlapping site information inherent for CTCF, we checked whether mean fold enrichment of overlapping CTCF sites is higher than non-overlapping sites between CTCF sites in E0-12 embryo, KC cells and S2 cells. Using both the Welch Two sample T-test and Wilcox test, the null hypothesis that true difference in means is equal to 0, was rejected with very high confidence (p-values < 1e-^16^ in all cases).

**Joint model analysis**

ChIP-chip data from multiple cell types was jointly analyzed with a probe by probe linear mixed model of the form:

*y_rh_ ~ μ + I_h_ + Χ_r_ + I_h_ x Χ_r_ + Π_r_ + ε_rh_*

Where *y* is the log2 transformed probe intensity, *μ* is the expected probe intensity, *I_h_* is the effect of immunoprecipitation on hybridization *h*, and *Χ_r_* is the effect of the cell type of prep *r*. *I_h_ x Χ_r_* represents the interaction effect between cell type and immunoprecipitation, e.g., caused by cell type specific binding sites. *Π* is the random effect due to cell prep effects and *ε* is the residual error. The model was fitted to each gene by residual maximum likelihood using the lmer function in the R package lme4 [13,14]. Fixed effect p-values were estimated by F-tests, as implemented in the aovlmer.fnc function in the languageR package [15]. -10*Log10 transformed p-values were smoothened with a 7 probe moving average. Probes with transformed, smoothened IP effects greater than 35 were called ‘significant.’ This threshold corresponded to a false discovery rate of less than 1%. Significant binding sites were identified as runs of significant probes spanning at least 130 bases. From such an initial seed, binding sites were greedily expanded to include all significant probes separated from the seed by less than 130 bases.

**Identification of active promoters**

The intersection of four datasets was used to identify unambiguously active promoters in *Drosophila melanogaster* embryos: (a) refseq annotated transcription start sites, (b) the presence of an H3K4Me3 enriched region within 250 and 750 bases up and downstream, respectively, (c) the presence of a PolII enriched region within 250 and 750 bases, and (c) exonic overlap with a region of significant total RNA signal. For the analyses of divergently or adjacently transcribed genes, active promoters were called based on an overlap of all 4 criteria. We identified active, alternatively transcribed promoters, based on an overlap of the first 3 criteria, as differentially active promoters could not be identified based on RNA signal from downstream exons. Due to the lack of PolII and RNA tiling data in S2 and Kc cell lines, active promoters were defined solely on the overlap of the first two categories.

**References**

1. Johnson WE, Li W, Meyer CA, Gottardo R, Carroll JS, et al. (2006) Model-based analysis of tiling-arrays for ChIP-chip. Proc Natl Acad Sci U S A 103: 12457-12462.

2. Li W, Carroll JS, Brown M, Liu S (2008) xMAN: extreme MApping of OligoNucleotides. BMC Genomics 9 Suppl 1: S20.

3. Schwartz YB, Kahn TG, Nix DA, Li XY, Bourgon R, et al. (2006) Genome-wide analysis of Polycomb targets in *Drosophila* melanogaster. Nat Genet 38: 700-705.

4. Parkinson H, Kapushesky M, Kolesnikov N, Rustici G, Shojatalab M, et al. (2009) ArrayExpress update--from an archive of functional genomics experiments to the atlas of gene expression. Nucleic Acids Res 37: D868-872.

5. Bolstad BM, Irizarry RA, Astrand M, Speed TP (2003) A comparison of normalization methods for high density oligonucleotide array data based on variance and bias. Bioinformatics 19: 185-193.

6. Ji H, Jiang H, Ma W, Johnson DS, Myers RM, et al. (2008) An integrated software system for analyzing ChIP-chip and ChIP-seq data. Nat Biotechnol 26: 1293-1300.

7. Day N, Hemmaplardh A, Thurman RE, Stamatoyannopoulos JA, Noble WS (2007) Unsupervised segmentation of continuous genomic data. Bioinformatics 23: 1424-1426.

8. Adryan B, Woerfel G, Birch-Machin I, Gao S, Quick M, et al. (2007) Genomic mapping of Suppressor of Hairy-wing binding sites in *Drosophila*. Genome Biol 8: R167.

9. Kent WJ (2002) BLAT--the BLAST-like alignment tool. Genome Res 12: 656-664.

10. Holohan EE, Kwong C, Adryan B, Bartkuhn M, Herold M, et al. (2007) CTCF genomic binding sites in *Drosophila* and the organisation of the bithorax complex. PLoS Genet 3: e112.

11. Eisen MB, Spellman PT, Brown PO, Botstein D (1998) Cluster analysis and display of genome-wide expression patterns. Proc Natl Acad Sci U S A 95: 14863-14868.

12. D'Haeseleer P (2005) How does gene expression clustering work? Nat Biotechnol 23: 1499-1501.

13. Bates D, Maechler M, Dai B (2008) lme4: Linear mixed-effects models using S4 classes. R package version 0.999375-27 ed.

14. R Development Core Team (2008) R: A language and environment for statistical computing. Vienna, Austria: R Foundation for Statistical Computing.

15. Baayen RH (2008) languageR: Data sets and functions with "Analyzing Linguistic Data: A practical introduction to statistics". R package version 0.953.

16. Parnell TJ, Viering MM, Skjesol A, Helou C, Kuhn EJ, et al. (2003) An endogenous suppressor of hairy-wing insulator separates regulatory domains in *Drosophila*. Proc Natl Acad Sci U S A 100: 13436-13441.

17. Golovnin A, Biryukova I, Romanova O, Silicheva M, Parshikov A, et al. (2003) An endogenous Su(Hw) insulator separates the yellow gene from the Achaete-scute gene complex in *Drosophila*. Development 130: 3249-3258.

18. Kellum R, Schedl P (1992) A group of scs elements function as domain boundaries in an enhancer-blocking assay. Mol Cell Biol 12: 2424-2431.

19. Belozerov VE, Majumder P, Shen P, Cai HN (2003) A novel boundary element may facilitate independent gene regulation in the Antennapedia complex of *Drosophila*. EMBO J 22: 3113-3121.

20. Matys V, Fricke E, Geffers R, Gossling E, Haubrock M, et al. (2003) TRANSFAC: transcriptional regulation, from patterns to profiles. Nucleic Acids Res 31: 374-378.

21. Zhao K, Hart CM, Laemmli UK (1995) Visualization of chromosomal domains with boundary element-associated factor BEAF-32. Cell 81: 879-889.

22. Emberly E, Blattes R, Schuettengruber B, Hennion M, Jiang N, et al. (2008) BEAF regulates cell-cycle genes through the controlled deposition of H3K9 methylation marks into its conserved dual-core binding sites. PLoS Biol 6: 2896-2910.

23. Blangy A, Leopold P, Vidal F, Rassoulzadegan M, Cuzin F (1991) Recognition of the CDEI motif GTCACATG by mouse nuclear proteins and interference with the early development of the mouse embryo. Nucleic Acids Res 19: 7243-7250.

24. MacArthur S, Li XY, Li J, Brown JB, Chu HC, et al. (2009) Developmental roles of 21 *Drosophila* transcription factors are determined by quantitative differences in binding to an overlapping set of thousands of genomic regions. Genome Biol 10: R80.
